# A Population Model of Folate-Mediated One-Carbon Metabolism

**DOI:** 10.3390/nu5072457

**Published:** 2013-07-05

**Authors:** Tanya M. Duncan, Michael C. Reed, H. Frederik Nijhout

**Affiliations:** 1 Department of Biology, Duke University, Durham, NC 27708, USA; E-Mail: tmk5@duke.edu; 2 Department of Mathematics, Duke University, Durham, NC 27708, USA; E-Mail: reed@math.duke.edu

**Keywords:** folate cycle, methionine cycle, mathematical model, population model, NHANES

## Abstract

*Background*: Previous mathematical models for hepatic and tissue one-carbon metabolism have been combined and extended to include a blood plasma compartment. We use this model to study how the concentrations of metabolites that can be measured in the plasma are related to their respective intracellular concentrations. *Methods*: The model consists of a set of ordinary differential equations, one for each metabolite in each compartment, and kinetic equations for metabolism and for transport between compartments. The model was validated by comparison to a variety of experimental data such as the methionine load test and variation in folate intake. We further extended this model by introducing random and systematic variation in enzyme activity. *Outcomes and Conclusions*: A database of 10,000 virtual individuals was generated, each with a quantitatively different one-carbon metabolism. Our population has distributions of folate and homocysteine in the plasma and tissues that are similar to those found in the NHANES data. The model reproduces many other sets of clinical data. We show that tissue and plasma folate is highly correlated, but liver and plasma folate much less so. Oxidative stress increases the plasma *S*-adenosylmethionine/*S*-adenosylhomocysteine (SAM/SAH) ratio. We show that many relationships among variables are nonlinear and in many cases we provide explanations. Sampling of subpopulations produces dramatically different apparent associations among variables. The model can be used to simulate populations with polymorphisms in genes for folate metabolism and variations in dietary input.

## 1. Introduction

The folate and methionine cycles play critical roles in cell metabolism with profound consequences for health and disease. Defects in the enzymes or insufficiencies in the B vitamins that act as cofactors and carbon carriers in these metabolic cycles have been associated with birth defects, certain cancers, cardiovascular disease and neurological disorders [1,2,3,4,5,6]. Among the critical enzymes in the folate and methionine cycles are thymidylate synthase, which provides the rate-limiting step for DNA synthesis [7] and the DNA methyl transferases (DNMTs) that regulate epigenetic control of gene transcription. In the methionine cycle, *S*-adenosylmethionine (SAM) is the universal methyl donor and is a substrate for over 150 methyl transferases [8]. The level of SAM and the *S*-adenosylmethionine/*S*-adenosylhomocysteine (SAM/SAH) ratio are thought to be good indicators of methylation capacity [9]. The methionine cycle, via cystathionine-β-synthase (CBS), also provides the first step in the synthesis of glutathione, the principal endogenous antioxidant [10,11]. Lowered levels of folate and elevated levels of homocysteine (Hcy) in the plasma are commonly used as indicators of insufficiency in these metabolic cycles and are used as biomarkers for oxidative stress, and for the risk of neurodegenerative disease, atherosclerosis, coronary heart disease, and birth defects [5,9,12].

The status of the folate and methionine cycles is typically assessed by measuring plasma levels of folate, homocysteine and the SAM/SAH ratio. Although plasma metabolites are well established as biomarkers of disease, an understanding of the mechanism of disease requires knowing metabolite levels in the cells, how these are affected by variation in genetics and nutrition, and exactly how that variation is reflected in their plasma values. Previously we developed a mathematical model of the methionine cycle in the liver and peripheral tissues, and of transport mechanisms between these compartments and the plasma, and we showed that under some circumstances the plasma levels of metabolites are not good indicators of their intracellular levels [13]. More importantly, by *in silico* experimentation, that model made it possible to investigate and explain the causes of those deviations and to explore specifically how differences in folate status, methionine input, and enzyme polymorphisms, affect the levels of intracellular and plasma metabolites.

In the present paper we expand our mathematical model in two ways. First, we incorporate the folate cycle into the liver and tissue compartments of the methionine cycle model, and account for the uptake of folate and its transport, as 5-methyltetrahydrofolate (5mTHF), between liver, tissue, and plasma compartments and its elimination in the urine. Second, we develop a version of the model that allows us to introduce stochastic variation in the activities of enzymes, transporters, and nutrient inputs, as well as systematic variation due to polymorphisms in the genes in this system. With this model we create large populations of virtual individuals with specific patterns of polymorphisms and variation in nutrition. We show that the plasma and tissue distributions of folate and homocysteine in this virtual population closely resemble those distributions in the NHANES studies. The population model also accurately reproduces distributions of plasma metabolites measured in populations with different genetic makeups. We use the population data to study and explain the associations between plasma and intracellular levels of metabolites. We show that the relationships among metabolite concentrations and reaction fluxes are often highly non-linear, which may help explain the variability and context-dependency of empirical data on the associations of biomarkers with disease outcomes.

## 2. Experimental Section

**Figure 1:**
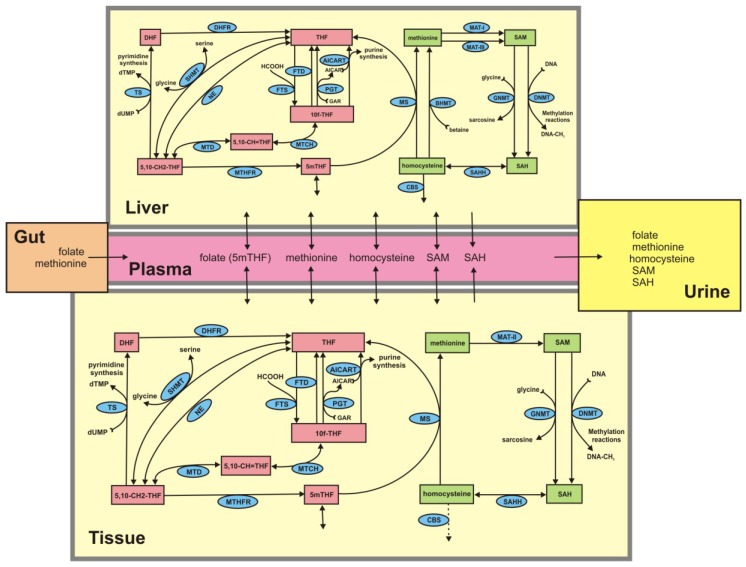
Structure of the metabolic system described by the model. Boxes are metabolites, ellipses contain the acronyms for enzymes, and arrows indicate the directions of flux. Full names of the metabolites and enzymes are in the Supplementary Materials.

We previously developed a mathematical model for intracellular methionine cycle kinetics in the liver and peripheral tissue compartments that contained input of substrates into the plasma, transport of methionine and folate between plasma and liver, plasma and tissues, and removal of metabolites by catabolism and excretion [13]. The tissue compartment refers to non-liver tissues and includes erythrocytes. In the present study we expand this model in two ways. First, we incorporate the folate cycle into the liver and peripheral tissue compartments, as outlined in [14,15,16], and add transport of SAM, SAH, and 5mTHF between the plasma, tissue, liver and urine. The metabolic reaction diagrams and transport directions are shown in Figure 1. Full names of all acronyms are in the Supplementary Materials.

The kinetics of the liver and tissue methionine and folate cycles are derived from [13,14,15,16,17]. The model consists of 26 differential equations that express the rates of change of the metabolites in Figure 1. Each of the differential equations is a mass balance equation expressing the time rate of change of the concentration of the particular metabolite as the sum of the rates at which it is being made minus the rates at which it is being consumed in biochemical reactions, plus or minus the net transport rates from or to other compartments. The differential equations, rate equations, transporter kinetics and justifications are given in the Supplementary Materials together with all parameter values and steady-state values. The model was implemented in MATLAB (Mathworks, Natick, MA, USA).

Second, we used this expanded model to create virtual populations by introducing stochastic variation in the activities of enzymes and input rates as follows. To simulate stochastic variation in enzyme activity, the *V*_max_ of each enzyme was multiplied by a number close to one taken from a log-normal distribution (using the location parameter μ = −0.09, and the scale parameter σ = 0.427, giving a mean of 1 and a standard deviation of 0.2). The log-normal distribution is widespread and probably the most common frequency distribution in biological systems [18]. In addition, we simulated genetic polymorphisms (for instance in thymidylate synthase (TS) and methylene-tetrahydrofolate reductase (MTHFR)) by using a random number generator to select two “alleles” from the known frequency distributions, using for each allele a *V*_max_ value that corresponds to the enzyme activity of that mutant (as given in [19]). In all cases the two alleles were assumed to act additively. For each set of random choices, the model was then run to equilibrium giving the metabolite concentrations and reaction fluxes for that virtual individual. We then followed this procedure many (1000–10,000) times to generate a population of virtual individuals, in which each “individual” had a different randomly generated set of enzyme activities. Statistical analyses were done using JMP Pro 9.0 (SAS Institute Inc., Cary, NC, USA).

## 3. Results

### 3.1. Experiments on Model Individuals

In order to be sure that the mathematical model represents the underlying physiology well, we conducted numerous *in silico* experiments, where the results could be compared to clinical studies in the literature. We discuss three of those experiments here.

**Figure 2:**
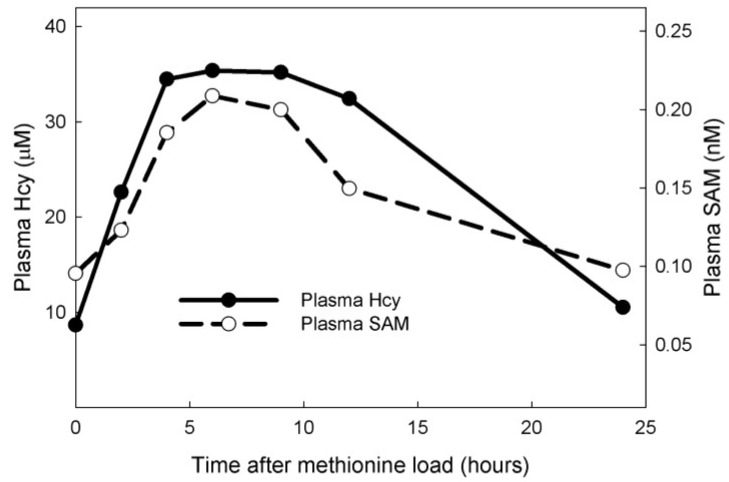
Model simulation of a methionine load test. The curves show the response of plasma homocysteine (Hcy) (filled circles) and *S*-adenosylmethionine (SAM) (open circles) to a pulse of methionine.

First, we conducted a methionine load test on the model. We added a pulse to the methionine input that raised the methionine in the plasma by a factor of 3 with a peak about 3 h after the dose. The exact formula for the methionine pulse is given in the Supplementary Materials. The model then computed the time courses of all the concentrations in the model and all the fluxes. In Figure 2 we show the model computations of plasma Hcy and plasma SAM, two variables that are commonly measured in the clinic. These curves correspond very well with those measured in [20].

**Figure 3:**
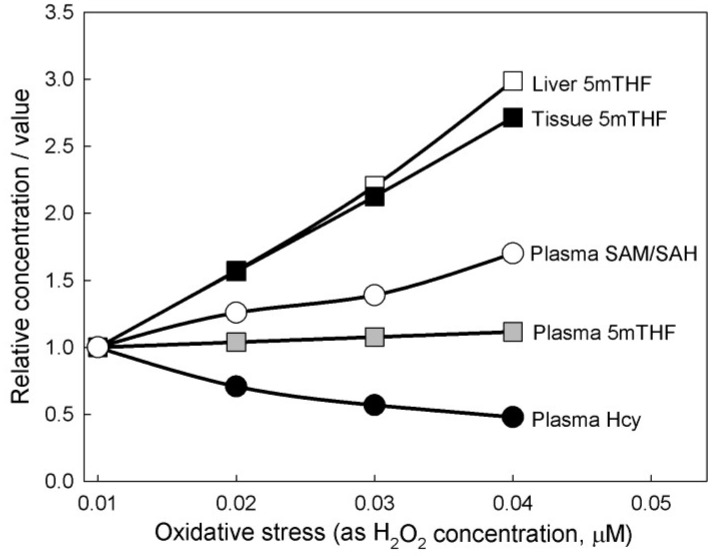
Effect of increasing oxidative stress. Model calculations show that increasing oxidative stress from normal (0.01 μM hydrogen peroxide (H_2_O_2_)) causes plasma Hcy to decrease, the plasma *S*-adenosylmethionine/*S*-adenosylhomocysteine (SAM/SAH) ratio to increase, and the tissue and liver concentrations of 5-methyltetrahydrofolate (5mTHF) to increase considerably. Thus oxidative stress creates a methyl trap. Plasma 5mTHF is unchanged and therefore is not a good indicator of oxidative stress.

Next, we examined the effects of oxidative stress on folate and methionine metabolism by raising the level of H_2_O_2_ in our model from 0.01 μM (normal) to 0.02, 0.03, and 0.04. The results can be seen in Figure 3. The most striking consequence is that 5mTHF in the liver and in the peripheral tissues increases about threefold at the highest level of oxidative stress. Thus, oxidative stress in our model creates a methyl trap wherein 5mTHF increases considerably and the substrates for the TS and AICART reactions decrease considerably, thereby inhibiting cell division. We note that although intracellular levels of 5mTHF increase dramatically, the plasma level changes only slightly, and thus plasma levels of 5mTHF are not a good biomarker of oxidative stress. Jill James [9,21] studied autistic children and found that they have high oxidative stress (higher reduced glutathione (GSSG) and lower glutathione (GSH)). She found, as we do, that Hcy decreases in the plasma compared to normal controls.

Experiments with this model and our GSH model [22] provide the reason why intracellular levels of 5mTHF increase substantially. Oxidative stress increases dramatically the intracellular concentration of GSSG, a substrate that inhibits MAT-I and MAT-III [23]. This lowers the flux around the methionine cycle. In addition oxidative stress stimulates the enzyme CBS, so more of the lowered flux is sent down the transsulfuration pathway. This lowers intracellular Hcy, which greatly decreases the flux in the MS reaction, thereby causing 5mTHF to build up.

**Figure 4:**
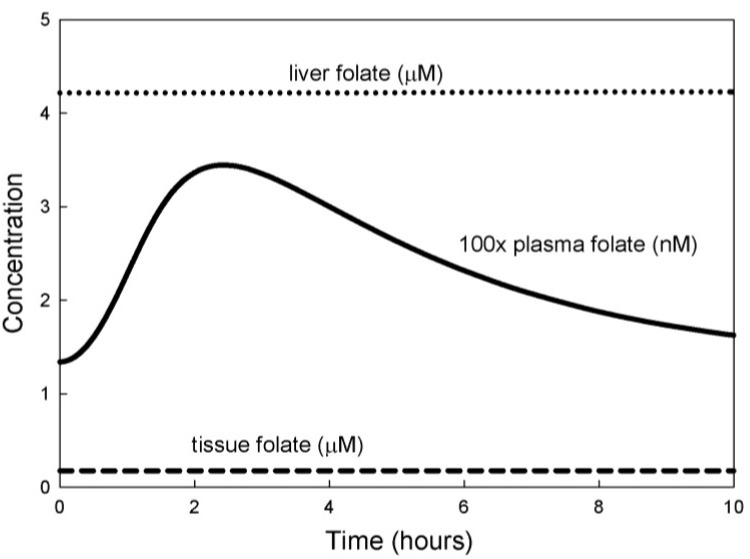
Response to a folate pulse. The curves show model computations of 5mTHF in the plasma, liver and tissue after a pulse of 5mTHF. Intracellular values do not change but plasma values rise almost 3-fold and take more than 8 h to return to steady-state.

Finally, we examined the effect on the model of an extra pulse of folate (5mTHF) input to the plasma such as one would get by taking a folate supplement. We did not attempt to model the difficult and controversial questions surrounding folate bioavailability [24,25,26], which involve breakdown in the gut and transfer to the plasma, but instead simply increased the input of 5mTHF to the plasma in pulsatile fashion. Most of the extra folate enters the plasma in the first two hours after the dose; the exact formula is given in the Supplementary Materials. Figure 4 shows that plasma folate increases rapidly, peaking at about two hours and then declines slowly so that it is almost back to normal after 10 h. The time course of plasma folate computed by the model is very similar to the clinical curves in [25,27]. Notice that a single supplemental dose of folate has no noticeable effect on liver or tissue folate (Figure 4). This corresponds with many clinical observations that plasma folate is quite variable, but liver and tissue folate change slowly on a much longer time scale. We also conducted experiments with the model (simulations not shown) that showed that liver and tissue folate have a half-life of about 90 days, corresponding to clinical observations [1].

### 3.2. Population Studies

We generated a virtual population of 10,000 individuals as outlined in Methods. In addition to stochastic variation in each enzyme, this virtual population contains polymorphisms for the genes for two enzymes: MTHFR (modeling the *677C* > *T* polymorphism; frequency of the T allele = 0.31; activity of enzyme CC = 100%, CT = 60%, TT = 30%) and TS (modeling the *1494del6* polymorphism; frequency of *−6bp* = 0.32; activity of enzyme +*6bp*/+*6bp* = 100%, +*6bp*/−*6bp* = 48%, −*6bp*/−*6bp* = 24%). A spreadsheet with all metabolite concentrations and reaction fluxes for these 10,000 individuals is deposited in the Supplementary Materials as DuncanPopulationData.xls. Data for the genotypic values of each enzyme for each individual in this virtual population are also given. We used this virtual population to generate several of the figures and tables discussed below.

#### 3.2.1. Comparison with NHANES Data

The mean plasma folate, tissue folate, and plasma Hcy concentrations for the individuals in the population model are shown in Table 1, together with NHANES postfortification data for 1999–2000 and 2001–2002 [28]. The model predicts folate and homocysteine levels within the ranges found by the NHANES studies.

**Table 1:** Comparison of model results to NHANES data.

Metabolite		*N*	Mean
Plasma folate *			
	Model ^1^ (post-fortification)	10,000	29.7 ± 0.2
	NHANES ^2,3^ (1999–2000)	3223	30.2 ± 0.7
	NHANES ^2,3^ (2001–2002)	3931	27.8 ± 0.5
Tissue folate *			
	Model ^1^ (post-fortification)	10,000	602 ± 2.15
	NHANES ^2,3^ (1999–2000)	3249	618 ± 0.11
	NHANES ^2,3^ (2001–2002)	3977	611 ± 0.9
Plasma Hcy **			
	Model ^1^ (post-fortification)	10,000	7.1 ± 0.03
	NHANES ^2,3^ (1999–2000)	3246	7.0 ± 0.01
	NHANES ^2,3^ (2001–2002)	3976	7.3 ± 0.01

* nmol/L; ^1^ values are averages ± SE; ^2^ NHANES analysis from [28]; ^3^ values are geometric means ± SE. ** μmol/L.

**Figure 5:**
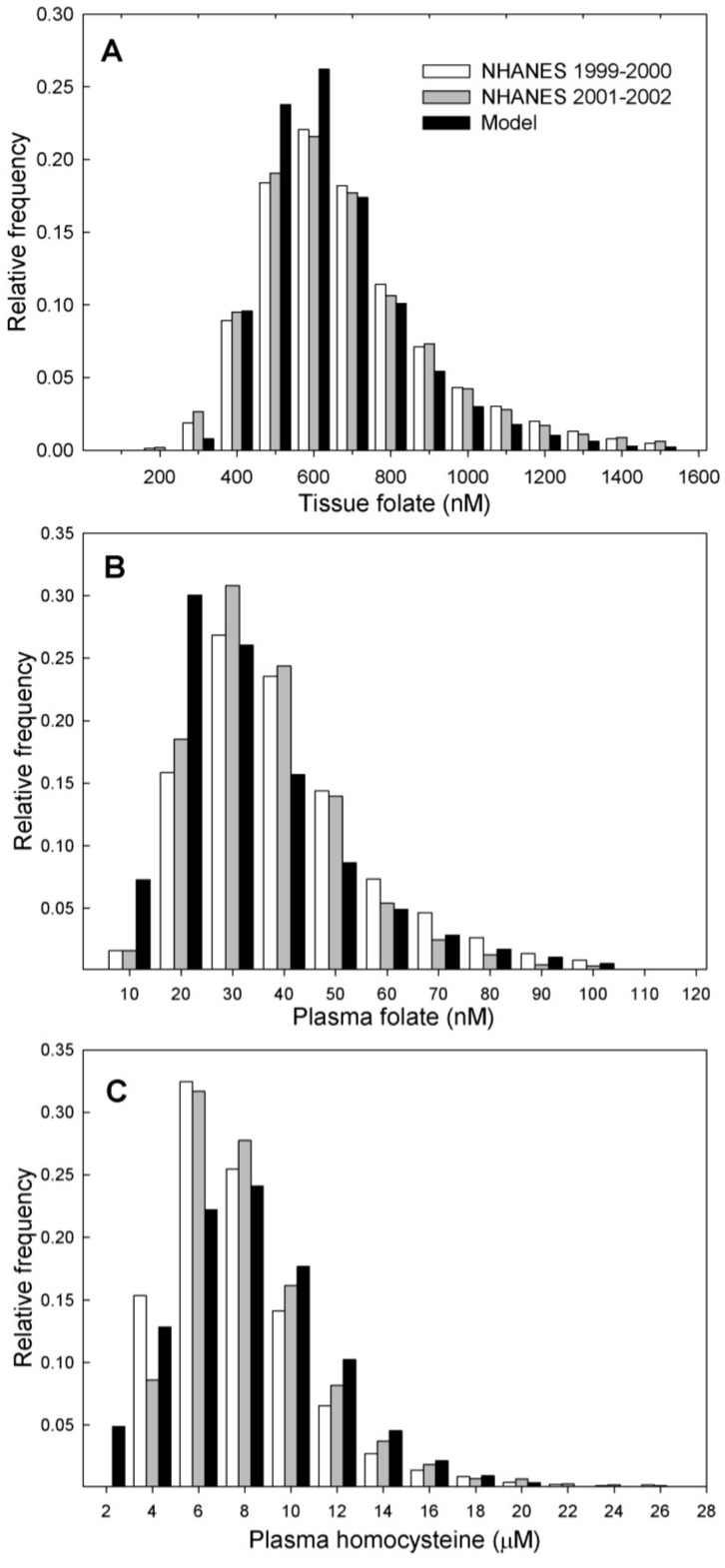
Frequency distributions of (**A**) tissue folate (**B**) plasma folate and (**C**) plasma Hcy found in two NHANES data sets are compared to distributions in the model population for In the model, population plasma folate is represented by 5mTHF and tissue folate is the sum of all folate derivatives.

The frequency distributions of individual tissue folate, plasma folate, and plasma Hcy levels for our virtual population model are shown in Figure 5, where they are compared to the distributions found in the NHANES studies. The tissue folate and plasma Hcy distributions are nearly identical to the NHANES data. In our model a higher frequency of individuals was found to have low plasma folate levels than reported in the NHANES studies. One possible explanation for this deviation is that individuals may have other sources of folate, such a gut microbiota, that are not included in our model. Another is that plasma folate levels are very sensitive to recent folate intake [29], making them quite variable (e.g., Figure 4), and this may account for the discrepancy. Real populations of course differ in accuracy of self-reporting dietary intake and in many uncontrolled and unknowable factors that might affect folate levels. Recently, it has been shown that the several different methods used in the NHANES studies to measure plasma folate levels generated large variation in the levels reported [30]. The NHANES data are thus subject to inaccuracies in measurement and incomplete knowledge of recent folate intake, yet the fit of the model results to these empirical data are very good.

#### 3.2.2. Plasma Hcy and 5mTHF

**Figure 6:**
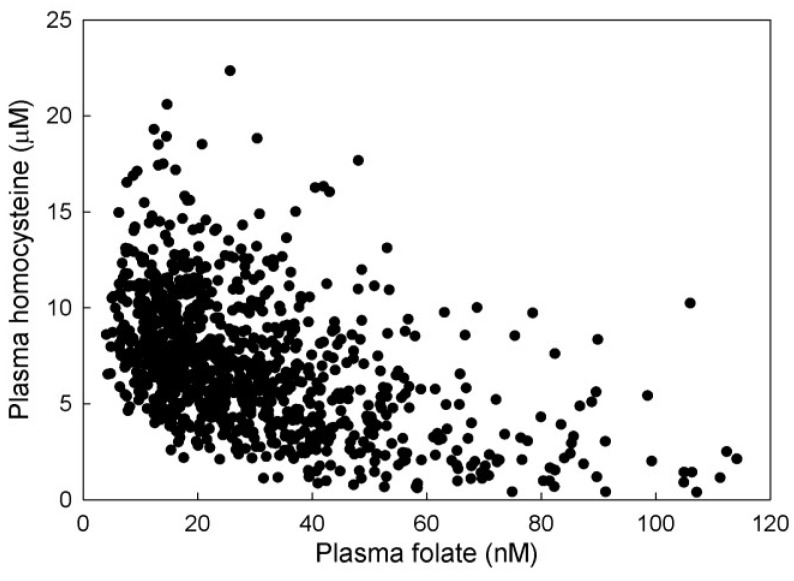
Scattergram of the relationship between plasma 5mTHF and plasma Hcy. Each point is an individual in a simulated population of 1000 virtual individuals.

**Figure 7:**
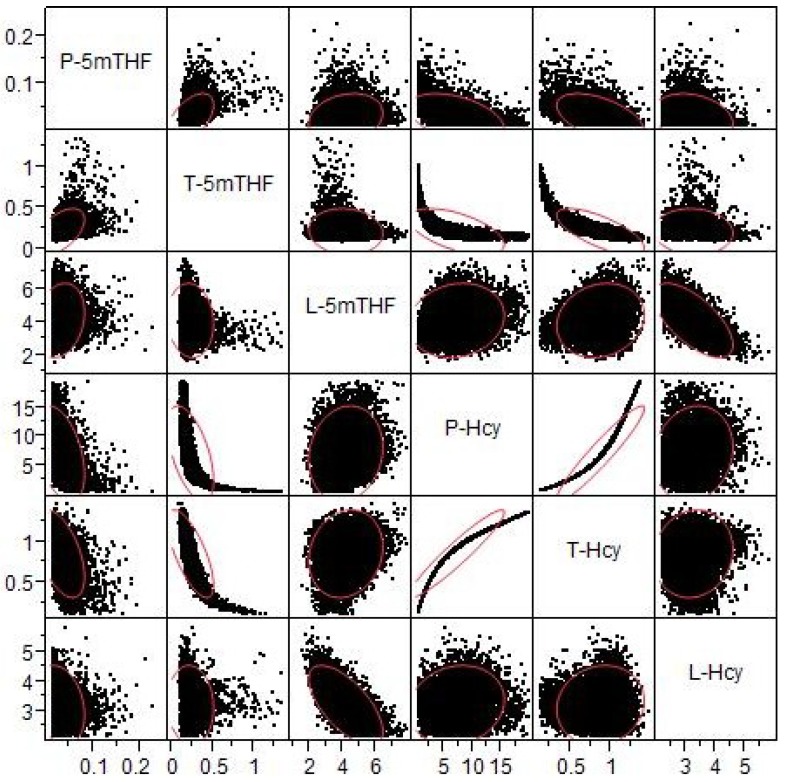
Scattergrams of relationships between 5mTHF and Hcy in plasma (P-), tissue (T-) and liver (L-). The diagonal gives the names of the metabolites; for each metabolite the column represents variation of the variable along the *x*-axis and the *y*-axes in the column show the variation of the different row-variables. Each graph contains 10,000 data points, one for each virtual individual. The red ellipses contain approximately 95% of the data points. Units of the axes are μM, except for P-5mTHF where they are nM.

The model results show that level of plasma Hcy is negatively correlated with plasma folate level (Figure 6), although the relationship is not linear. This corresponds well with the relationship found by Selhub *et al.* [31]. At high plasma folate levels, these authors found somewhat higher plasma Hcy than we see in our simulations, which may be due to the fact that we are simulating a post-fortification population. The correlations between 5mTHF and Hcy levels in the three compartments are shown in Figure 7. Plasma Hcy is strongly correlated with tissue Hcy but not with liver Hcy. This indicates that variation in plasma values is determined almost entirely by variation in tissue levels of Hcy. This is because the volume of the tissue compartment is much larger than that of the plasma and the liver, and because the tissue is a net exporter of Hcy whereas the liver imports Hcy from the plasma and remethylates it to methionine. We found that the levels of liver homocysteine and liver 5mTHF are well correlated. The relationship between tissue Hcy and tissue 5mTHF is highly nonlinear (Figure 7), indicating that one is not a good predictor of the other.

#### 3.2.3. Methylation Capacity

**Figure 8:**
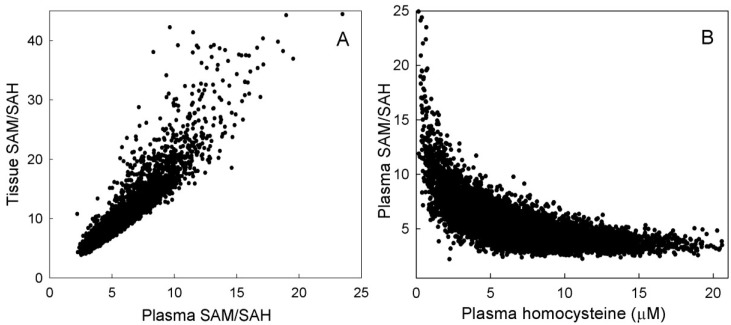
Model scattergram for plasma *versus* tissue SAM/SAH ratio (**A**) and for plasma Hcy *versus* the plasma SAM/SAH ratio (**B**). Plasma and tissue SAM/SAH are well-correlated. Plasma Hcy is not a good predictor of plasma SAM/SAH.

The SAM/SAH ratio is commonly used as an index of cellular methylation capacity [12,32]. A decrease in the SAM/SAH ratio can be due to a decrease in SAM, the universal methyl donor, or an increase in SAH, which is a general inhibitor of methyl transfer reactions [33,34]. Melynk *et al.* [12] found a positive relationship between plasma SAM/SAH and lymphocyte SAM/SAH, suggesting that plasma values are a good indicator of intracellular values. Our model results show a positive relationship between plasma SAM/ SAH and tissue SAM/SAH (Figure 8A), similar to that found by Melnyk *et al.* [12], as well as a negative association between plasma SAM/ SAH and plasma Hcy (Figure 8B), likewise similar to the findings of Melnyk *et al.* [12].

**Figure 9:**
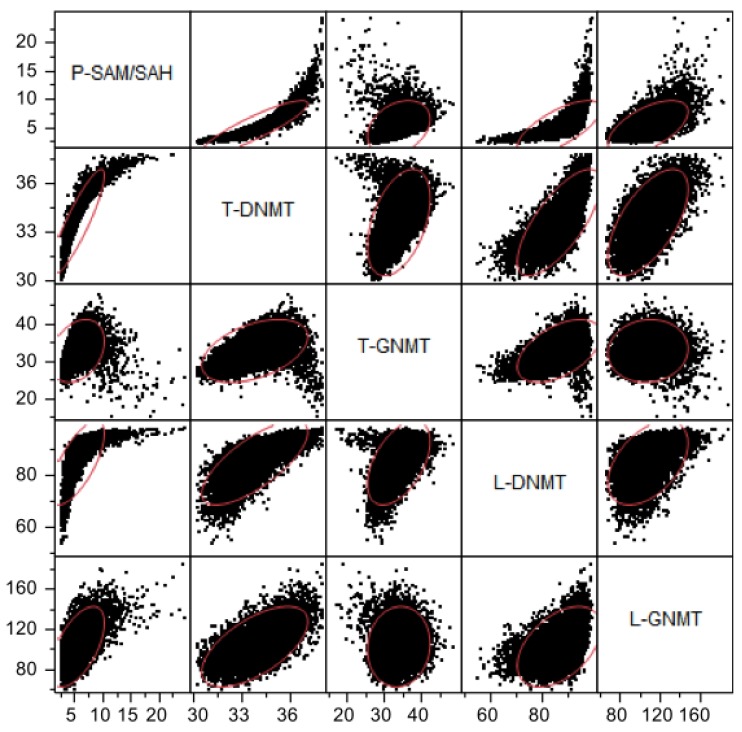
Scattergrams of the relationships between plasma (P-) SAM/SAH ratio and the DNA-methyltransferase (DNMT) and glycine *N*-methyltransferase (GNMT) reaction fluxes in liver (L-) and tissues (T-). Each graph contains 10,000 data points; the red ellipses contain approximately 95% of the data. Units of axes are μM/h.

In our model we have two methyltransferases, DNMT and GNMT, and assume that the sum of the flux through these two enzymes is a measure of the total methylation rate. The model reveals that the maximum flux through the DNMT reactions is associated with low to moderate plasma SAM/SAH ratios and saturates at medium to high ratios (Figure 9). Flux through the GNMT reactions, by contrast, is positively correlated with the plasma SAM/SAH ratio at low to medium values, but decreases when the SAM/SAH ratio becomes very high (Figure 9). The reason for the decline in methylation capacity at a very high SAM/SAH ratio is that high concentrations of SAM inhibit MTHFR, which reduces the availability of 5mTHF, and this slows down the methionine synthase reaction and flux around the methionine cycle.

#### 3.2.4. Interaction among Mutations

**Figure 10:**
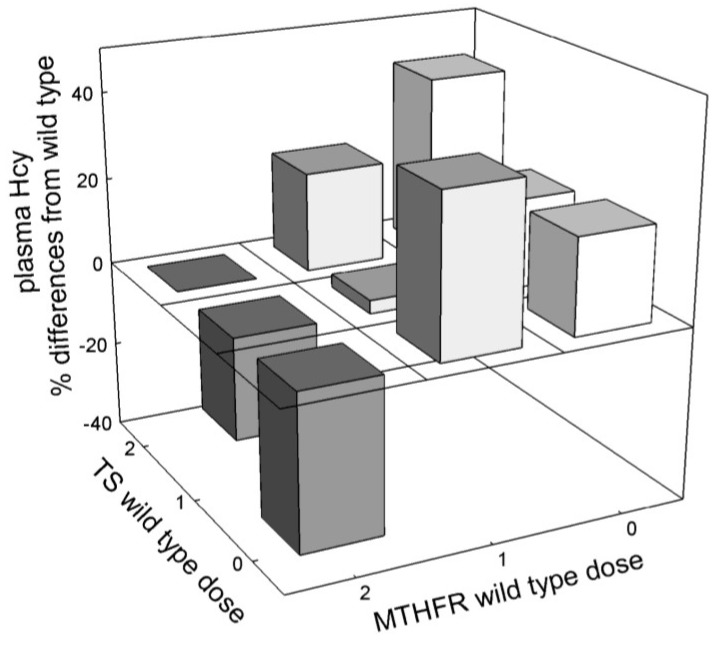
Mean values of plasma Hcy as a function of 5,10-methylenetetrahydrofolate reductase (MTHFR) and thymidylate synthase (TS) genotypes in a population of 10,000 virtual individuals. The basis for comparison is the double homozygous wild-type (2,2). The TS polymorphism decreases plasma Hcy and the MTHFR polymorphism increases plasma Hcy. However their joint effects are not additive.

Our virtual population has polymorphisms in MTHFR and TS. The activities of the enzymes for homozygotes and heterozygotes for these mutations and the allele frequencies are described in Section 3.2. The C677T polymorphism of MTHFR has been shown to be associated with an increased plasma Hcy, whereas the 1494del6 polymorphism of TS is associated with a decreased plasma Hcy [19,35,36]. In a sizable population many individuals will carry both mutations, either in the heterozygous or homozygous condition. Our virtual population allowed us to examine the population-wide interactions among these mutations. Their joint effects on plasma Hcy are shown in Figure 10, which illustrates the mean deviations from the mean wild-type phenotype for each genotype. It is clear that the effect of gene dosage is not always monotonic, and the interactions among the two genes are not additive. The reduced function allele of TS appears to have a stronger effect on plasma Hcy than that of MTHFR.

#### 3.2.5. Correlations with Extreme Plasma Hcy Values

**Figure 11:**
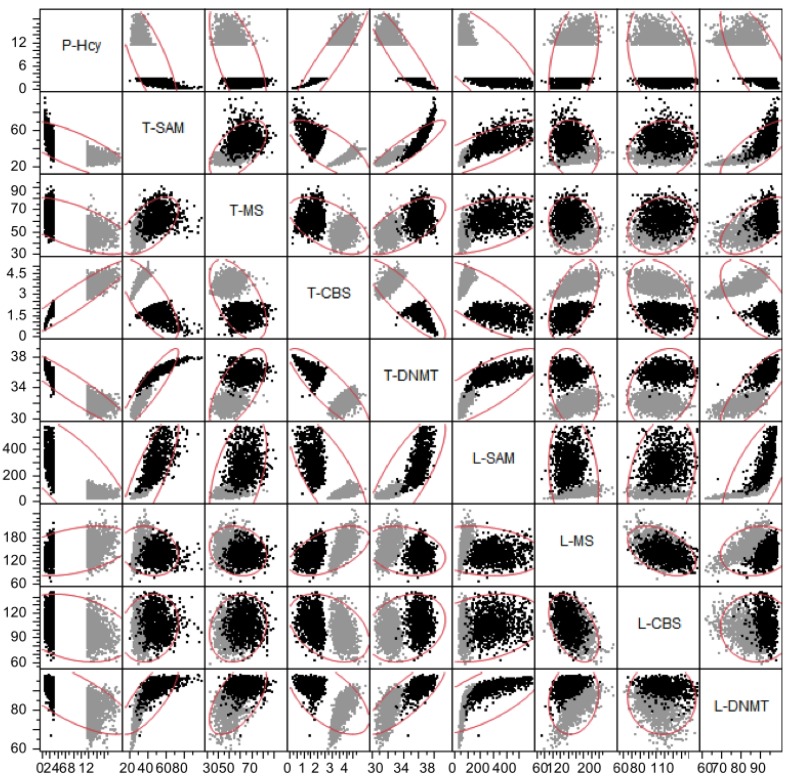
Scattergrams for various metabolite concentrations and reaction rates in virtual individuals with high plasma Hcy (gray) and low plasma Hcy (black). The 1000 individuals with the highest plasma Hcy and the 1000 individuals with the lowest plasma Hcy were selected from a population of 10,000 virtual individuals. The scattergrams allow one to assess whether the relationship between other variables differ in the two sub-populations. Units of the axes are μM for concentrations and μM/h for reaction rates.

From our database population of 10,000 we selected the 1000 individuals with the highest plasma Hcy concentrations, and the 1000 individuals with the lowest plasma Hcy concentrations in order to study which internal variables (metabolite concentrations, enzyme fluxes) were best associated with these extremes. Scattergrams of selected pairwise combinations are shown in Figure 11. In general, the values of tissue metabolites and enzyme fluxes are better correlated with plasma Hcy than are those in the liver. This is because the peripheral tissue is a much larger compartment. The strongest correlations with plasma Hcy were the tissue CBS and DNMT reactions (Figure 11). The correlation with CBS activity is positive, as one would expect, since higher levels of Hcy will drive that reaction faster. There is no relationship, however, with tissue MS activity, presumably because MS also requires 5mTHF as a co-substrate; higher concentrations of Hcy would drive the MS reaction faster and thus draw down 5mTHF. There is a negative relationship between plasma Hcy and tissue DNMT rate (Figure 11), which is due to the negative association of Hcy and tissue SAM, which is the substrate for the DNMT reaction. Most other enzymes and metabolites show a weak correlation with plasma Hcy, at least in these simple pairwise comparisons.

#### 3.2.6. Nonlinear Relationships

**Figure 12:**
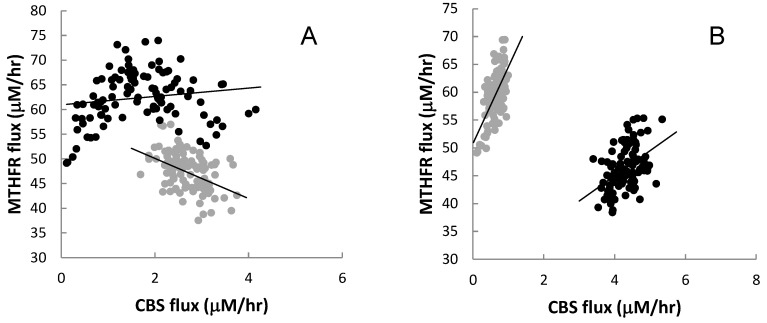
Estimated relationships between CBS and MTHFR fluxes based on selective data from the same population of 10,000 virtual individuals. Panel (**A**) shows 100 individuals with the highest (black) and 100 with the lowest (gray) plasma folate. Panel (**B**) shows 100 individuals with the highest (black) and 100 with the lowest (gray) plasma Hcy. For each sub-population the slope of the regression estimating the relationship between CBS and MTHFR fluxes is quite different. Lines are least-square regressions.

The results illustrated in Figure 6, Figure 7, Figure 8, Figure 9, Figure 11 show that the relationships among metabolites and reaction fluxes in this system can be very nonlinear. Indeed, a global analysis of relationships throughout the system modeled here shows that almost half the pairwise relationships among metabolites and reaction fluxes are nonlinear (see also Figures S2 and S3 in Supplementary Materials). This finding implies that observed correlations among variables in the system will be sensitive to the values of other (possibly unmeasured) variables. Sampling a sub-population with particular characteristics, for instance, can produce dramatically different apparent associations among variables. Figure 12 is an example of the relationship between fluxes through the CBS and MTHFR reactions for four different subsamples of the population. Each subpopulation has a different relationship even though the underlying mechanism is exactly the same for all.

## 4. Discussion

We have developed a mathematical model for whole-body one-carbon metabolism and the transport of metabolites between the plasma, liver and peripheral tissues. We developed two versions of the model: an individual model in which we studied the time-dependence of variation in cellular metabolism due to variation in nutrient and vitamin input, and a population model in which we studied the effects of variation in enzyme activities, nutrient input and genetic polymorphisms on the distribution of steady-state values of metabolite concentrations and metabolic fluxes.

A methionine load test with the individual model produces plasma profiles of Hcy and SAM that are closely similar to those found empirically. The individual model also shows that a pulse of folate input changes the plasma folate significantly but has little effect on the tissue and liver folate levels. Prolonged exposure to altered folate input levels is required to substantially change the liver and tissue values [13]. We also used the individual model to examine the effects of increased oxidative stress on metabolite levels and reaction fluxes. Oxidative stress causes substantial changes in tissue and liver 5mTHF, which are not reflected in plasma values. The model allowed us to explain the causal pathway by which oxidative stress produces this effect.

We used the model to produce a population of 10,000 virtual individuals with stochastic variation in enzyme activities and polymorphisms in MTHFR and TS. The frequency distribution of tissue folate and of plasma folate and homocysteine was nearly identical to that found in the NHANES studies, which suggests that the amount and distribution of stochastic variation in our virtual population fairly resembled that of a US population. The model population distributions of plasma and tissue SAM/SAH ratios, and the relationship between plasma Hcy, folate and SAM/SAH are likewise similar to those found in human population studies.

For obvious reasons, most biomarkers are measured in the blood or in the urine and then one assumes that these values are a good representation of what is happening in the liver or the tissues. Our model gives a way of assessing this assumption. P-5mTHF is not very correlated to T-5mTHF or L-5mTHF (Figure 7). P-Hcy is tightly correlated with T-Hcy (even though the relationship is nonlinear), but P-Hcy is quite uncorrelated with L-Hcy (Figure 7). P-SAM/SAH is reasonably well correlated with T-SAM/SAH (Figure 8A). P-SAM is tightly correlated to T-SAM but only moderately coupled to L-SAM (scatterplots not shown). In general, we have found that tissue values and plasma values are often well correlated but plasma values and liver values are not. This is not surprising since the volume of the tissues in our model is much larger than the volume of the liver. Nevertheless, these results show that plasma-tissue and plasma-liver correlations may differ considerably for different variables and that it is always risky to assume that the plasma measurements represent well either tissue or liver values.

The population model allows us to partition a population by genotype and/or phenotype and investigate the associations of intracellular metabolites and reaction fluxes and of plasma metabolites with each different condition. Partitioning a population by individuals with the highest and lowest plasma Hcy values allowed us to determine which other pairwise relationships are different for the two populations. For example, in all the pairwise scatterplots of CBS with other variables in Figure 11, the high and low Hcy populations fall in two separate clouds. The reason is easy to understand. Plasma Hcy is highly correlated with tissue Hcy (see Figure 7). Thus, if plasma Hcy is high (low), then tissue Hcy will be high (low) and will drive the CBS flux at a high (low) rate. On the other hand, some of the scatter plots of tissue MS flux *versus* other variables show separate populations (T-MS *vs.* T-CBS), some show partially overlapping populations (T-MS *vs.* L-MS), and some show almost completely overlapping populations (L-MS *vs.* L-CBS). In each case, one can use the reaction diagram (Figure 1) and model experiments to figure out why these scatterplot results are true. Thus, the virtual population allows one to investigate the behavior of all concentrations and fluxes, and correlations between them in selected subpopulations.

When one does such an investigation, one can sometimes find surprising results. In Figure 12A we saw that MTHFR flux was positively correlated with CBS flux in the high folate populations but negatively correlated with CBS flux in the low folate population. And in Figure 12B we saw that the positive relationship of MTHFR flux to CBS flux has a very different slope in the high Hcy population as compared to the low Hcy population. This is a good reminder that one has to be very careful to choose a “random” population if one expects to infer universal relationships between variables.

Perhaps the most striking result obtained from our population studies is that many of the scatterplots are highly nonlinear; that is, there is no linear relationship between the variables that captures the essential features of the data. Consider, for example, the scatterplots of T-5mTHF *vs.* P-Hcy in Figure 7, of P-SAM/SAH *vs.* L-DNMT in Figure 9, or T-GNMT *vs.* T-DNMT in Figure 9. In one sense such outcomes are not surprising since almost all the velocities in the reaction diagram (Figure 1) are (highly) nonlinear functions of the current values of various concentrations. On the other hand, when one computes correlations, it is tempting to assume that the linear regression line expresses a real relationship between the variables. These scatterplots are strong evidence that caution should be exercised in making such simplifying assumptions.

## 5. Conclusions

1. The deterministic model is a useful tool for studying the casual relationships between changes in different metabolite and biomarker levels.

2. The virtual population model allows one to study interesting relationships suggested by the NHANES studies or other population studies, because in our model one can study the correlations among *all* the variables in the system.

3. One needs to take great care before assuming that changes in plasma levels of metabolites will be simple reflections of changes in liver levels of the same metabolites.

4. Selecting random populations is crucial to population studies, because correlations between variables may be very different in different non-random subpopulations.

## Supplementary Files

Supplementary File 1 Supplementary Material (DOCX, 4552 KB)

Supplementary File 2 Compatibility mode (XLS, 20042 KB)
