# Supplementary material for: A Population Model of Folate-Mediated One-Carbon Metabolism
Source: Nutrients. 2013 Jul 5;5(7):2457–74. doi: 10.3390/nu5072457 (PMC3738981; doi:10.3390/nu5072457)
Supplement: Supplementary File 1 — Supplementary Material (DOCX, 4552 KB) [file nutrients-05-02457-s001.docx]

**Supplementary Material**

**Figure S1.** Reaction diagram of the three-compartment model. Rectangular boxes contain the acronyms of substrates and blue ellipses contain the acronyms of enzymes. For full names, see Tables S1 and S2.


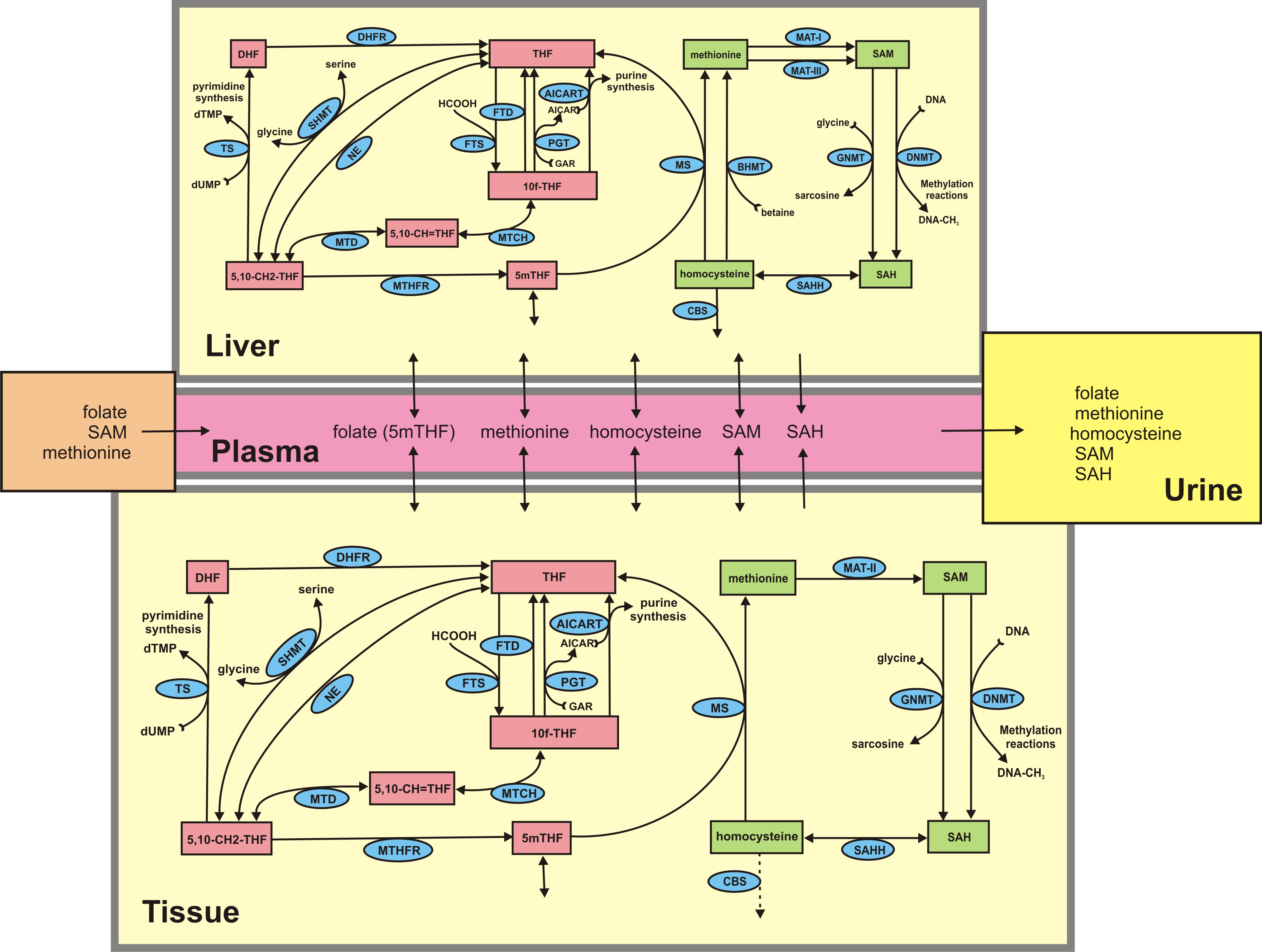


**Details of the Mathematical Model**

The model consists of 26 differential equations that express the rates of change of the metabolites in Figure 1. The mathematical model merges and enhances our previously published whole body models of the methionine cycle [1] and the folate cycle [2–5]. Each of the differential equations in the model is a mass balance equation; the time rate of change of the particular metabolite equals the sum of the rates at which it is being made minus the rates it is being consumed in biochemical reactions, plus or minus the net transport rates from or to other compartments.

In order to display the differential equations coherently, we have chosen notation for the variables and reaction rates that is both more uniform and sparse than some notation commonly in use. For example, the concentration of methionine in the liver is denoted lMet instead of the usual [liverMet]. Our notation is described in Part A, below. In Part B, we give the differential equations, which are written in terms of reaction, transport and removal rates, and contain terms to account for the relative sizes of the three compartments. In Part C, the kinetic formulas and constants for these reaction and transport rates are given with justifications. Part D describes metabolite input and removal from
the system.

**Part A. Notation**

*A.1. Names and Acronyms*

The names of the enzymes indicated by acronyms in Figure 1 are given in Table S1.

**Table S1.** Enzyme names and acronyms.

| **Folate Cycle** | |
| --- | --- |
| AICAR(T) | aminoimidazolecarboxamide ribonucleotide (transferase) |
| FTD | 10-formyltetrahydrofolate dehydrogenase |
| FTS | 10-formyltetrahydrofolate synthase |
| MTCH | 5,10-methylenetetrahydrofolate cyclohydrolase |
| MTD | 5,10-methylenetetrahydrofolate dehydrogenase |
| MTHFR | 5,10-methylenetetrahydrofolate reductase |
| TS | thymidylate synthase |
| SHMT | serine hydroxymethyltransferase |
| PGT | phosphoribosyl glycinamidetransformalase |
| NE | nonenzymatic interconversion of THF and 5,10-CH2-THF |
| DHFR | dihydrofolate reductase |
| **Methionine Cycle** | |
| MAT-I | methionine adenosyl transferase I |
| MAT-II | methionine adenosyl transferase II |
| MAT-III | methionine adenosyl transferase III |
| GNMT | glycine *N*-methyltransferase |
| DNMT | DNA-methyltransferase |
| SAHH | *S*-adenosylhomocysteine hydrolase |
| CBS | cystathionine β-synthase |
| MS | methionine synthase |
| BHMT | betaine-homocysteine methyltransferase |

We use three letter acronyms or abbreviations for most metabolites (Table S2). In the equations, these acronyms have a prefix of l, t, p, or u to indicate the compartment, liver, tissue, plasma or urine, respectively.

**Table S2.** Names and acronyms of metabolites.

| **Folate Cycle** | |
| --- | --- |
| 5mTHF | 5-methyltetrahydrofolate |
| THF | tetrahydrofolate |
| 10fTHF | 10-formyltetrahydrofolate |
| DHF | dihydrofolate |
| CH2-THF | 5,10-methylenetrahydrofolate |
| CHF | 5,10-methenyltetrahydrofolate |

**Table S2.** *Cont*.

| **Methionine Cycle** | |
| --- | --- |
| Met | methionine |
| SAM | *S*-adenosylmethionine |
| SAH | *S*-adenosylhomocysteine |
| Hcy | homocysteine |

*A.2. Constants*

**Table S3.** Names and values of constants (concentrations in µM, time in h), and size ratios of the three compartments.

| **Name** | **Value** | **Description** |
| --- | --- | --- |
| [H_2_O_2_]_norm_ | 0.01 | normal steady state intracellular hydrogen peroxide |
| [H_2_O_2_] | 0.01 | intracellular hydrogen peroxide (differs in some experiments) |
| Folin | 0.0046 | hourly input of folate (varies in population) |
| Metin | 100 | hourly input of methionine |
| [GAR] | 10 ^1^ | glycinamide ribonucleotide concentration |
| [AICAR] | 2.1 ^1^ | aminoimidazolecarboxamide ribonucleotide concentration |
| [NADPH] | 50 ^1^ | glycine concentration |
| [GLY] | 1850 ^1^ | serine concentration |
| [HCOOH] | 900 ^1^ | formate concentration |
| [H_2_CO] | 500 ^1^ | formaldehyde concentration |
| [DUMP] | 20 ^1^ | deoxyuridine monophosphate concentration |
| k_lp_ | 0.625 | liver to plasma size ratio |
| k_pl_ | 1.6 | plasma to liver size ratio |
| k_tp_ | 7.5 | tissue to plasma size ratio |
| k_pt_ | 0.133 | plasma to tissue size ratio |

^1^ Literature references found in [6].

*A.3. Steady State Values*

**Table S4.** Mean (lower 95% mean, upper 95% mean) of steady-state values of metabolite concentrations in the liver, plasma and tissues in a pre-fortified population of 10,000 individuals.

| **Compartment** | **Metabolite** | **Model** | **Data** | **Reference** |
| --- | --- | --- | --- | --- |
| Plasma | Hcy (µM) | 8.66 (8.59,8.72) | 8.7 ± 0.1 | [7] |
|  | SAM (nM) | 93.03 (92.49,93.57) | 35–118 | [8] |
|  | SAH (nM) | 23.61 (22.56,22.65) | 9.6–38.7 | [8] |
|  | 5mTHF (nM) | 16.67 (16.29,17.04) | 12.1 ± 0.3 | [7] |
|  | Met (µM) | 34.40 (34.17,34.62) | 24.1 ± 4.7 | [9] |

**Table S4.** *Cont*.

| Liver |  |  |  |  |
| --- | --- | --- | --- | --- |
|  | Hcy (µM) | 3.58 (3.56,3.59) | 3.63 ± 0.89 | [10] |
|  | SAM (µM) | 84.66 (82.91,86.41) | 60–90 | [10] |
|  | SAH (µM) | 15.40 (15.31,15.49) | 10–15 | [10] |
|  | Met (µM) | 78.03 (77.58,78.49) | 72.6 ± 12.5 | [11] |
|  | ^1^ Total Folate(µM) | 21.03 (20.30,21.30) |  |  |
|  | 5mTHF (µM) | 3.64 (3.62,3.66) | 4.6–8 | [12] |
|  | THF (µM) | 6.74 (6.68,6.81) | 1.8–6.8 | [12] |
|  | DHF(µM) | 0.009 (0.009,0.009) | 0.023–0.12 | [12] |
|  | CH2-THF (µM) | 0.98 (0.96,1.00) | 1–2.5 | [12] |
|  | CHF (µM) | 1.00 (0.99,1.01) | 2.7–11.2 | [12] |
|  | 10fTHF (µM) | 8.65 (8.48,8.82) | 1–16.5 | [12] |
| Tissue |  |  |  |  |
|  | Hcy (µM) | 0.95 (0.95,0.96) | 0.76–1.12 | [10] |
|  | SAM (µM) | 29.98 (29.83,30.13) | 19–50 | [10] |
|  | SAH (µM) | 4.38 (4.37,4.39) | 3.4–6.7 | [10] |
|  | Met (µM) | 55.71 (55.24,56.18) | 63 ± 13.0 | [13]  [7] |
|  | ^1^ Total Folate(µM) | 0.45(0.44,0.45) | 391 ± 0.5 |  |
|  | 5mTHF (µM) | 0.19 (0.19,0.19) |  |  |
|  | THF (µM) | 0.23 (0.22,0.23) |  |  |
|  | DHF(µM) | 0.0019 (0.0019,0.0019) |  |  |
|  | CH2-THF (µM) | 0.012(0.012,0.012) |  |  |
|  | CHF (µM) | 0.002 (0.002,0.002) |  |  |
|  | 10fTHF(µM) | 0.018(0.017,0.018) |  |  |

^1^ Total Folate = 5mTHF + THF +DHF + CH2-THF + CHF + 10fTHF

**Part B. The Equations**

*B.1. Velocity Notation*

We denote the velocity of a reaction (in μM/h) by a capital *V*, with a subscript indicating the acronym for the enzyme that catalyzes the reaction. For example, the velocity of the methionine synthase (MS) reaction is denoted by *V*_MS_. For the transport of metabolites into and out of compartments the velocity and direction of the transport reaction (in µM/h) is indicated by a capital *V*, with a subscript indicating the acronym for the metabolite being transported, and the first and last letter surrounding the metabolite indicates its movement. For example, transport of methionine into the liver from the plasma, or out of the liver and into the plasma, are denoted by *V*_pMetl_ and *V*_lMetp_, respectively.

B.2. Compartment Size

The relative size of each compartment was calculated from the human tissue mass balance data in [14], their Table 21. We calculated the relative size of the plasma, liver, and metabolically active tissue to be 4%, 2.5%, and 30% of the total body mass, respectively. When transferring metabolites from one compartment to the other we multiplied the concentration in the receiving compartment by its size relative to that of the delivering compartment. In the equations, this relative size was expressed as a constant k, with a subscript with the first letter being the delivering compartment and the second the receiving compartment.

*B.3. The Differential Equations*

| $\frac{d[pHcy]}{\mathrm{dt}}=k_{\mathrm{lp}}V_{\mathrm{lHcyp}}+ k_{\mathrm{tp}}V_{\mathrm{tHcyp}}{- V}_{\mathrm{pHcyl}}- V_{\mathrm{pHcyt}}- V_{\mathrm{pHcy}}+ V_{\mathrm{uHcyp}}$ |
| --- |
| $\frac{d[lHcy]}{\mathrm{dt}}={k_{\mathrm{pl}}V_{\mathrm{pHcyl}}{- V}_{\mathrm{lHcyp}}+V_{\mathrm{SAHH}}(\left[ \mathrm{SAH} \right],\left[ \mathrm{Hcy} \right])- V_{\mathrm{CBS}}(\left[ \mathrm{Hcy} \right],\left[ \mathrm{SAM} \right],[SAH])-V_{\mathrm{MS}}(\left[ 5mTHF \right],\left[ \mathrm{Hcy} \right],\left[ H_{2}O_{2} \right]_{norm})- V_{\mathrm{BHMT}}([Hcy}],\left[ \mathrm{SAM} \right],\left[ \mathrm{SAH} \right],\left[ H_{2}O_{2} \right],\left[ H_{2}O_{2} \right]_{norm})$ |
| $\frac{d[tHcy]}{\mathrm{dt}}={{k_{\mathrm{pt}}V}_{\mathrm{pHcyt}}{- V}_{\mathrm{tHcyp}}+V_{\mathrm{SAHH}}(\left[ \mathrm{SAH} \right],\left[ \mathrm{HCY} \right])- V_{\mathrm{CBS}}(\left[ \mathrm{Hcy} \right],\left[ \mathrm{SAM} \right],[SAH])-V_{\mathrm{MS}}(\left[ 5mTHF \right],\left[ \mathrm{Hcy} \right],\left[ H_{2}O_{2} \right],\left[ H_{2}O_{2} \right]_{norm})}$ |
| $\frac{d[pSAM]}{\mathrm{dt}}= {k_{\mathrm{lp}}V}_{\mathrm{lSAMp}}+ {k_{\mathrm{tp}}V}_{\mathrm{tSAMp}}-V_{\mathrm{pSAMu}}$ |
| $\frac{d[lSAM]}{\mathrm{dt}}= -V_{\mathrm{lSAMp}}{+ V}_{\mathrm{MATI}}\left( \left[ \mathrm{MET} \right],\left[ \mathrm{SAM} \right],\left[ H_{2}O_{2} \right],\left[ H_{2}O_{2} \right]_{norm} \right)+ V_{\mathrm{MATIII}}\left( \left[ \mathrm{MET} \right],\left[ \mathrm{SAM} \right],\left[ H_{2}O_{2} \right],\left[ H_{2}O_{2} \right]_{norm} \right) - V_{\mathrm{GNMT}}(\left[ \mathrm{SAM} \right],\left[ \mathrm{SAH} \right],\left[ 5mTHF \right])-V_{\mathrm{DNMT}}(\left[ \mathrm{SAM} \right],\left[ \mathrm{SAH} \right])$ |
| $\frac{d[tSAM]}{\mathrm{dt}}= -V_{\mathrm{tSAMp}}+ V_{\mathrm{MATII}}\left( \left[ \mathrm{MET} \right],\left[ \mathrm{SAM} \right],\left[ H_{2}O_{2} \right],\left[ H_{2}O_{2} \right]_{norm} \right)- V_{\mathrm{GNMT}}\left( \left[ \mathrm{SAM} \right],\left[ \mathrm{SAH} \right],\left[ 5mTHF \right] \right) -V_{\mathrm{DNMT}}(\left[ \mathrm{SAM} \right],\left[ \mathrm{SAH} \right])$ |
| $\frac{d[pSAH]}{\mathrm{dt}}={k_{\mathrm{lp}}V}_{\mathrm{lSAHp}}+ k_{\mathrm{tp}}V_{\mathrm{tSAHp}}- V_{\mathrm{pSAHu}}$ |
| $\frac{d[lSAH]}{\mathrm{dt}}= -V_{\mathrm{lSAHp}}+ V_{\mathrm{GNMT}}\left( \left[ \mathrm{SAM} \right],\left[ \mathrm{SAH} \right],\left[ 5mTHF \right] \right)+ V_{\mathrm{DNMT}}\left( \left[ \mathrm{SAM} \right],\left[ \mathrm{SAH} \right] \right) -V_{\mathrm{SAHH}}(\left[ \mathrm{SAH} \right],\left[ \mathrm{Hcy} \right])$ |
| $\frac{d[tSAH]}{\mathrm{dt}}= -V_{\mathrm{tSAHp}}+ V_{\mathrm{GNMT}}\left( \left[ \mathrm{SAM} \right],\left[ \mathrm{SAH} \right],\left[ 5mTHF \right] \right)+ V_{\mathrm{DNMT}}\left( \left[ \mathrm{SAM} \right],\left[ \mathrm{SAH} \right] \right) -V_{\mathrm{SAHH}} (\left[ \mathrm{SAH} \right],\left[ \mathrm{Hcy} \right])$ |
| $\frac{d\left[ p5mTHF \right]}{\mathrm{dt}}=Folin- V_{p5mTHFl}-V_{p5mTHFt} + V_{ldiff5mTHF} + V_{tdiff5mTHF}- V_{p5mTHFu}$ |
| $\frac{d[l5mTHF]}{\mathrm{dt}}={k_{\mathrm{pl}}\times V}_{p5mTHFl}- V_{ldiff5mTHF}+V_{\mathrm{MTHFR}}\left( \left[ CH2-THF \right],\left[ \mathrm{NADPH} \right],\left[ \mathrm{SAM} \right],\left[ \mathrm{SAH} \right] \right)- V_{\mathrm{MS}}(\left[ 5mTHF \right],\left[ \mathrm{HCY} \right],\left[ H_{2}O_{2} \right],\left[ \mathrm{ssH}_{2}O_{2} \right])$ |
| $\frac{d[t5mTHF]}{\mathrm{dt}}={k_{\mathrm{pt}}\times V}_{p5mTHFt}- V_{tdiff5mTHF}+V_{\mathrm{MTHFR}}\left( \left[ CH2-THF \right],\left[ \mathrm{NADPH} \right],\left[ \mathrm{SAM} \right],\left[ \mathrm{SAH} \right] \right)- V_{\mathrm{MS}}(\left[ 5mTHF \right],\left[ \mathrm{HCY} \right],\left[ H_{2}O_{2} \right],\left[ \mathrm{ssH}_{2}O_{2} \right])$ |
| $\frac{d\left[ p5mTHF \right]}{\mathrm{dt}}=Folin- V_{l5mTHFp}-V_{t5mTHFp} + V_{tflux5mTHF} + V_{lflux5mTHF}- V_{p5mTHFu}$ |
| $\frac{d[l5mTHF]}{\mathrm{dt}}={k_{\mathrm{pl}}V}_{p5mTHFl}- V_{lflux5mTHF}- V_{l5mTHF}+V_{\mathrm{MTHFR}}\left( \left[ CH2-THF \right],\left[ \mathrm{NADPH} \right],\left[ \mathrm{SAM} \right],\left[ \mathrm{SAH} \right] \right)- V_{\mathrm{MS}}(\left[ 5mTHF \right],\left[ \mathrm{Hcy} \right],\left[ H_{2}O_{2} \right],\left[ H_{2}O_{2} \right]_{norm})$ |
| $\frac{d[t5mTHF]}{\mathrm{dt}}={k_{\mathrm{pt}}V}_{p5mTHFt}- V_{tflux5mTHF}- V_{t5mTHF}+V_{\mathrm{MTHFR}}\left( \left[ CH2-THF \right],\left[ \mathrm{NADPH} \right],\left[ \mathrm{SAM} \right],\left[ \mathrm{SAH} \right] \right)- V_{\mathrm{MS}}(\left[ 5mTHF \right],\left[ \mathrm{Hcy} \right],\left[ H_{2}O_{2} \right],\left[ H_{2}O_{2} \right]_{norm})$ |
| $\frac{d[lTHF]}{\mathrm{dt}}=V_{\mathrm{FTD}}(\left[ 10fTHF \right])+ V_{\mathrm{MS}}(\left[ 5mTHF \right],\left[ \mathrm{Hcy} \right],\left[ H_{2}O_{2} \right],\left[ H_{2}O_{2} \right]_{norm})+ V_{\mathrm{PGT}}(\left[ 10fTHF \right],\left[ \mathrm{GAR} \right])+V_{\mathrm{AICAR}\left( T \right)}(\left[ 10fTHF \right],\left[ \mathrm{AICAR} \right])- V_{\mathrm{FTS}}(\left[ \mathrm{THF} \right],\left[ \mathrm{HCOOH} \right],\left[ 10fTHF \right])- V_{\mathrm{SHMT}}(\left[ \mathrm{SER} \right],\left[ \mathrm{THF} \right],\left[ \mathrm{GLY} \right],\left[ CH2-THF \right]{)-V}_{\mathrm{NE}}\left( \left[ \mathrm{THF} \right],\left[ H2\mathrm{CO} \right],\left[ CH2-THF \right] \right)+ V_{\mathrm{DHFR}}\left( \left[ \mathrm{DHF} \right],\left[ \mathrm{NADPH} \right] \right)- V_{\mathrm{lTHF}}$ |
| $\frac{d[tTHF]}{\mathrm{dt}}=V_{\mathrm{FTD}}(\left[ 10fTHF \right])+ V_{\mathrm{MS}}(\left[ 5mTHF \right],\left[ \mathrm{Hcy} \right],\left[ H_{2}O_{2} \right],\left[ H_{2}O_{2} \right]_{norm})+ V_{\mathrm{PGT}}(\left[ 10fTHF \right],\left[ \mathrm{GAR} \right])+V_{\mathrm{AICAR}\left( T \right)}(\left[ 10fTHF \right],\left[ \mathrm{AICAR} \right])- V_{\mathrm{FTS}}(\left[ \mathrm{THF} \right],\left[ \mathrm{HCOOH} \right],\left[ 10fTHF \right])- V_{\mathrm{SHMT}}(\left[ \mathrm{SER} \right],\left[ \mathrm{THF} \right],\left[ \mathrm{GLY} \right],\left[ CH2-THF \right]{)-V}_{\mathrm{NE}}(\left[ \mathrm{THF} \right],\left[ H2\mathrm{CO} \right],\left[ CH2-THF \right])+ V_{\mathrm{DHFR}}(\left[ \mathrm{DHF} \right],\left[ \mathrm{NADPH} \right]) {-V}_{\mathrm{tTHF}}$ |
| $\frac{d[lDHF]}{\mathrm{dt}}=V_{\mathrm{TS}}(\left[ \mathrm{DUMP} \right],\left[ CH2-THF \right])-V_{\mathrm{DHFR}}(\left[ \mathrm{DHF} \right],\left[ \mathrm{NADPH} \right])$ |
| $\frac{d\left[ \mathrm{tDHF} \right]}{\mathrm{dt}}=V_{\mathrm{TS}}\left( \left[ \mathrm{DUMP} \right],\left[ CH2-THF \right] \right)-V_{\mathrm{DHFR}}\left( \left[ \mathrm{DHF} \right],\left[ \mathrm{NADPH} \right] \right)$ |
| $\frac{d\left[ lCH2-THF \right]}{\mathrm{dt}} =V_{\mathrm{SHMT}} \left( \left[ \mathrm{SER} \right],\left[ \mathrm{THF} \right],\left[ \mathrm{GLY} \right],\left[ CH2-THF \right] \right)+ V_{\mathrm{NE}}\left( \left[ \mathrm{THF} \right],\left[ H2\mathrm{CO} \right],\left[ CH2-THF \right] \right)- V_{\mathrm{TS}}(\left[ \mathrm{DUMP} \right],\left[ CH2-THF \right])-V_{\mathrm{MTHFR}}(\left[ CH2-THF \right],\left[ \mathrm{NADPH} \right],\left[ \mathrm{SAM} \right],[SAH])- V_{\mathrm{MHD}}(\left[ CH2-THF \right],\left[ \mathrm{CHF} \right])$ |
| $\frac{d\left[ tCH2-THF \right]}{\mathrm{dt}} =V_{\mathrm{SHMT}} \left( \left[ \mathrm{SER} \right],\left[ \mathrm{THF} \right],\left[ \mathrm{GLY} \right],\left[ CH2-THF \right] \right)+ V_{\mathrm{NE}}\left( \left[ \mathrm{THF} \right],\left[ H2\mathrm{CO} \right],\left[ CH2-THF \right] \right)- V_{\mathrm{TS}}(\left[ \mathrm{DUMP} \right],\left[ CH2-THF \right])-V_{\mathrm{MTHFR}}(\left[ CH2-THF \right],\left[ \mathrm{NADPH} \right],\left[ \mathrm{SAM} \right],[SAH])- V_{\mathrm{MHD}}(\left[ CH2-THF \right],\left[ \mathrm{CHF} \right])$ |
| $\frac{d[lCHF]}{\mathrm{dt}}= V_{\mathrm{MHD}}\left( \left[ CH2-THF \right],[CHF] \right)-V_{\mathrm{MCH}}\left( \left[ \mathrm{CHF} \right],\left[ 10fTHF \right] \right)$ |
| $\frac{d[tCHF]}{\mathrm{dt}}= V_{\mathrm{MHD}}\left( \left[ CH2-THF \right],[CHF] \right)-V_{\mathrm{MCH}}\left( \left[ \mathrm{CHF} \right],\left[ 10fTHF \right] \right)$ |
| $\frac{d[l10fTHF]}{\mathrm{dt}}=V_{\mathrm{MCH}}(\left[ \mathrm{CHF} \right],\left[ 10fTHF \right])+ V_{\mathrm{FTS}}(\left[ \mathrm{THF} \right],\left[ \mathrm{HCOOH} \right],\left[ 10fTHF \right])- V_{\mathrm{PGT}}(\left[ 10fTHF \right],\left[ \mathrm{GAR} \right])-V_{\mathrm{AICAR}\left( T \right)}(\left[ 10fTHF \right],\left[ \mathrm{AICAR} \right])- V_{\mathrm{FTD}}(10fTHF)]$ |
| $\frac{d[t10fTHF]}{\mathrm{dt}}=V_{\mathrm{MCH}}(\left[ \mathrm{CHF} \right],\left[ 10fTHF \right])+ V_{\mathrm{FTS}}(\left[ \mathrm{THF} \right],\left[ \mathrm{HCOOH} \right],\left[ 10fTHF \right])- V_{\mathrm{PGT}}(\left[ 10fTHF \right],\left[ \mathrm{GAR} \right])-V_{\mathrm{AICAR}\left( T \right)}(\left[ 10fTHF \right],\left[ \mathrm{AICAR} \right])- V_{\mathrm{FTD}}(10fTHF)]$ |
| $\frac{d[pMet]}{\mathrm{dt}}= Metin +{k_{\mathrm{lp}}V}_{\mathrm{lMetp}}+k_{\mathrm{tp}}V_{\mathrm{tMetp}}{-V}_{\mathrm{pMetl}}- V_{\mathrm{pMett}}-V_{\mathrm{pMetu}}$ |
| $\frac{d\left[ \mathrm{lMet} \right]}{\mathrm{dt}}={{k_{\mathrm{pl}}V}_{\mathrm{pMetl}}{- V}_{\mathrm{lMetp}}+V_{\mathrm{BHMT}}\left( \left[ \mathrm{Hcy} \right],\left[ \mathrm{SAM} \right],\left[ \mathrm{SAH} \right],\left[ H_{2}O_{2} \right],\left[ H_{2}O_{2} \right]_{norm} \right)+}V_{\mathrm{MS}}\left( \left[ 5mTHF \right],\left[ \mathrm{Hcy} \right],\left[ H_{2}O_{2} \right],\left[ H_{2}O_{2} \right]_{norm} \right) - V_{\mathrm{MATI}}(\left[ \mathrm{MET} \right],\left[ SAM,\left[ H_{2}O_{2} \right],\left[ H_{2}O_{2} \right]_{norm} \right])-V_{\mathrm{MATIII}}(\left[ \mathrm{MET} \right],\left[ \mathrm{SAM} \right],\left[ H_{2}O_{2} \right],\left[ H_{2}O_{2} \right]_{norm})$ |
| $\frac{d\left[ \mathrm{tMet} \right]}{\mathrm{dt}}={{k_{\mathrm{pt}}V_{\mathrm{pMett}}}{- V}_{\mathrm{tMetp}} +}V_{\mathrm{MS}}\left( \left[ 5mTHF \right],\left[ \mathrm{Hcy} \right],\left[ H_{2}O_{2} \right],\left[ H_{2}O_{2} \right]_{norm} \right)-V_{\mathrm{MATII}}(\left[ \mathrm{MET} \right],\left[ \mathrm{SAM} \right],\left[ H_{2}O_{2} \right],\left[ H_{2}O_{2} \right]_{norm})$ |
| $\frac{d[uHcy]}{\mathrm{dt}}= V_{\mathrm{pHcyu}}$ |

**Part C. Kinetics**

*C.1. Oxidative Stress*

Intracellular hydrogen peroxide levels (H_2_O_2_) and reduced glutathione (GSSG) play a role in regulating enzyme velocities in the methionine cycle. Hydrogen peroxide inhibits MS and BHMT, and activates
CBS [4,15,16]. GSSG inhibits MAT-I, MAT-II, MAT-III [17,18]. To add inhibition by H_2_O_2_ to our enzyme reactions, we multiplied the reaction velocity by the term

| $\frac{K_{i}+{[H_{2}O_{2}]}_{norm}}{K_{i}+[H_{2}O_{2}]}$ |
| --- |

where $K_{i}$ is the scaling constant, and [H_2_O_2_]_norm_ is the concentration at steady-state. Since the H_2_O_2_ is in the denominator, the reaction velocity decreases as the concentration of H_2_O_2_ increases. We chose this format so that the velocity of the reaction at steady-state would remain the same once we added the inhibition. This allows us to study the effects of H_2_O_2_ changes on the system. We were unable to find kinetic data for the inhibitions by H_2_O_2_, so we chose our scaling constants to be the value of steady-state intracellular H_2_O_2_ concentration so that the effects of the inhibitions would be nearly linear. MAT-I, MAT-II and MAT-III are inhibited indirectly via reduced glutathione (GSSG), which accumulates under oxidative stress. GSSG does not occur in the present model, so we used our model for glutathione synthesis kinetics [4] to calculate the effective scaling constant of H_2_O_2_ on MAT-I and MAT-III.

For enzyme activation by H_2_O_2_, we used a similar approach. We multiplied the reaction velocity by the term:

| $\frac{K_{a}+[H_{2}O_{2}]}{K_{a}+{[H_{2}O_{2}]}_{norm}}$ |
| --- |

Note that here the H_2_O_2_ concentration is in the numerator, and so as the concentration of H_2_O_2_ increases, so does the velocity of the reaction. Again, the multiplier is one at steady-state.

*C.2. Enzyme Kinetics*

Folate Cycle

Some of the reactions in the Folate Cycle are unidirectional with one substrate, for example *V*_FTD_. We assume that their dependence on their substrate has Michaelis-Menten form:

| $\text{V}=\frac{V_{\max}[metabolite]}{K_{m}+[metabolite]}$ |
| --- |

Other reactions, for example *V*_SAHH_, *V*_MCH_, and *V*_MHD,_ are reversible Michaelis-Menten with one substrate in each term. Reactions with two substrates, for example *V*_AICAR(T)_, *V*_TS_, *V*_DHFR_, *V*_PGT_, and *V*_MS,_ are modeled by random order Michaelis-Menten kinetics and have the form:

| $V= \frac{V_{\max}\left[ metabolite1 \right][metabolite2]}{(K_{m,1}+\left[ metabolite1 \right])(K_{m,2}+\left[ metabolite2 \right])}$ |
| --- |

*V*_SHMT_ is assumed to have reversible random-order Michaelis-Menten kinetics with two substrates in each term. For all these velocities the form is clear and the *K*_m_ and *V*_max_ values appear in Table S5, below, along with references.

**Table S5.** Parameter values for enzymes in the folate cycle (*K*_m_ in μM, *V*_max_ in μM/h).

| **Enzyme** | **Compartment** | **Parameter** | **Model** | **Literature ^1^** |
| --- | --- | --- | --- | --- |
| AICAR(T) | Liver | $K_{m}^{10fTHF}$ | 5.9 | 5.9–50 |
|  |  | $K_{m}^{AICAR}$ | 100 | 10–100 |
|  |  | *V*_max_ | 81000 |  |
|  | Tissue | $K_{m}^{10fTHF}$ | 5.9 |  |
|  |  | $K_{m}^{AICAR}$ | 50 |  |
|  |  | *V*_max_ | 90000 |  |
| DHFR | Liver | $K_{m}^{DHF}$ | 0.5 | 0.12–1.9 |
|  |  | $K_{m}^{NADPH}$ | 4 | 0.3–5.6 |
|  |  | *V*_max_ | 10000 |  |
|  | Tissue | $K_{m}^{DHF}$ | 0.5 |  |
|  |  | $K_{m}^{NADPH}$ | 4 |  |
|  |  | *V*_max_ | 11250 |  |
| FTD | Liver | $K_{m}^{10fTHF}$ | 20 | 0.9 |
|  |  | *V*_max_ | 15400 |  |
|  | Tissue | $K_{m}^{10fTHF}$ | 20 |  |
|  |  | *V*_max_ | 9520 |  |
| FTS | Liver | $K_{m}^{THF}$ | 1 | 0.1–600 |
|  |  | $K_{m}^{HCOOH}$ | 43 | 8–1000 |
|  |  | V_max_ | 400 |  |
|  | Tissue | $K_{m}^{THF}$ | 3 |  |
|  |  | $K_{m}^{HCOOH}$ | 43 |  |
|  |  | *V*_max_ | 2500 |  |

**Table S5.** *Cont.*

| MTCH | Liver | $K_{m}^{CHF}$ | 250 | 4–250 |
| --- | --- | --- | --- | --- |
|  |  | V_max_ | 1600000 |  |
|  |  | $K_{m}^{10fTHF}$ | 100 | 20–450 |
|  |  | *V*_max_ | 20000 |  |
|  | Tissue | $K_{m}^{CHF}$ | 250 |  |
|  |  | *V*_max_ | 8000000 |  |
|  |  | $K_{m}^{10fTHF}$ | 100 |  |
|  |  | *V*_max_ | 200000 |  |
| MTD^2^ | Liver | $K_{m}^{CH2-THF}$ | 2 | 2–5 |
|  |  | *V*_max_ | 200000 |  |
|  |  | $K_{m}^{CHF}$ | 10 | 1–10 |
|  |  | *V*_max_ | 594000 |  |
|  | Tissue | $K_{m}^{CH2-THF}$ | 2 |  |
|  |  | *V*_max_ | 200000 |  |
|  |  | $K_{m}^{CHF}$ | 10 |  |
|  |  | *V*_max_ | 5940000 |  |
| PGT | Liver | $K_{m}^{10fTHF}$ | 4.9 | 4.9–58 |
|  |  | $K_{m}^{GAR}$ | 520 | 520 |
|  |  | *V*_max_ | 16200 |  |
|  | Tissue | $K_{m}^{10fTHF}$ | 4.9 |  |
|  |  | $K_{m}^{GAR}$ | 520 |  |
|  |  | *V*_max_ | 2592000 |  |
| SHMT^3^ | Liver | $K_{m}^{SER}$ | 600 | 350–1300 |
|  |  | $K_{m}^{THF}$ | 50 | 45–300 |
|  |  | *V*_max_ | 40000 |  |
|  |  | $K_{m}^{GLY}$ | 3000 | 3000–10,000 |
|  |  | $K_{m}^{CH2-THF}$ | 3200 | 3200–10,000 |
|  |  | *V*_max_ | 2500000 |  |
|  | Tissue | $K_{m}^{SER}$ | 600 |  |
|  |  | $K_{m}^{THF}$ | 50 |  |
|  |  | *V*_max_ | 4000 |  |
|  |  | $K_{m}^{GLY}$ | 3000 |  |
|  |  | $K_{m}^{CH2-THF}$ | 3200 |  |
|  |  | *V*_max_ | 250000 |  |
| TS | Liver | $K_{m}^{DUMP}$ | 6.3 | 5–37 |
|  |  | $K_{m}^{CH2-THF}$ | 14 | 10–45 |
|  |  | *V*_max_ | 5000 |  |
|  | Tissue | $K_{m}^{DUMP}$ | 6.3 |  |
|  |  | $K_{m}^{CH2-THF}$ | 14 |  |
|  |  | *V*_max_ | 90000 |  |

^1^ Literature references can be found in [3]. ^2^ Positive direction is from CH2-THF to CHF.
^3^ Positive direction is from THF to CH2-THF.

**NE.** The kinetics of the non-enzymatic reaction between THF and CH2-THF are taken to be mass action.

| *V_NE_* = *k*_1_[THF] − *k*_2_[CH2-THF] |
| --- |

The rate constants for the tissue are *k*_1_ = 1.004 and *k*_2_ = 0.01, and for the liver *k*_1_ = 1.001 and *k*_2_ = 0.01.

Methionine Cycle

**MAT-I**: The MAT-I kinetics are from [19], Table 1, and we take *V*_max_ = 257.79 μM/h and *K_m_* = 41. The inhibition by SAM was derived by non-linear regression on the data from [19], Figure 5. The last factor represents the inhibition of MAT-I by oxidative stress, with *K_i_*=35, see the discussion above.

| $V_{\mathrm{MATI}}=\left( 0.23+0.8e^{-0.0026[SAM]} \right) \left( \frac{V_{max}\left[ Met \right]}{K_{m}+ \left[ Met \right]} \right) \left( \frac{K_{i}+{[H_{2}O_{2}]}_{norm}}{K_{i}+[H_{2}O_{2}]} \right)$ |
| --- |

**MAT-II**: The methionine dependence of the MAT-II kinetics is from [19], and we take *V*_max_ = 178.47 μM/h and *K_m_* = 50. The inhibition by SAM was derived by non-linear regression on the data from [19], Figure 5. The last factor represents the inhibition of MAT-II by oxidative stress, with *K_i_* = 35, see the discussion above.

| $V_{\mathrm{MATII}}=\left( 0.15+0.83e^{-0.013[SAM]} \right) \left( \frac{V_{max}\left[ Met \right]}{K_{m}+ \left[ Met \right]} \right) \left( \frac{K_{i}+{[H_{2}O_{2}]}_{norm}}{K_{i}+[H_{2}O_{2}]} \right)$ |
| --- |

**MAT-III**. The methionine dependence of the MAT-III kinetics is from [20], Figure 5, fitted to a Hill equation with *V*_max_ = 56.67 μM/h, *K_m_* = 300. The activation by SAM is from [19], Figure 5, fitted to a Hill equation with *K_a_* = 360. The last factor represents the inhibition of MAT-III by oxidative stress, with *K_i_*=66, see the discussion above.

| $V_{\mathrm{MATIII}}=\left( \frac{V_{max}{[Met]}^{1.21}}{K_{m}+ {[Met]}^{1.21}} \right)\left( 1+\frac{7.2\left[ SAM \right]^{2}}{{{(K_{a})}^{2}+ [SAM]}^{2}} \right) \left( \frac{K_{i}+{[H_{2}O_{2}]}_{norm}}{K_{i}+[H_{2}O_{2}]} \right)$ |
| --- |

**GNMT:** The first term of the GNMT reaction is standard Michaelis-Menten with tissue *V*_max_ = 125.49 and liver *V*_max_ = 300.30 μM/h, and *K_m_* = 63 [21]. The second term is product inhibition by SAH
from [22] with *K_i_* = 18. The third term, the long-range inhibition of GNMT by 5mTHF, was derived by non-linear regression on the data of [23], and scaled so that it equals 1 when the 5mTHF concentration is 4.28 µM and 0.18 µM in the liver and tissue, respectively. The constant A is 4.63 and 0.52 in the liver and tissue, respectively.

| $V_{\mathrm{GNMT}}= \left( \frac{V_{max}\left[ SAM \right]}{K_{m}+ \left[ SAM \right]} \right)\left( \frac{1}{1+ \frac{[SAH]}{K_{i}}} \right)\left( \frac{A}{0.35+[5mTHF]} \right)$ |
| --- |

**DNMT:** The DNA methylation reaction is given as a uni-reactant scheme with SAM as substrate. That is, the substrates for methylation are assumed constant. Their variation can be modeled by varying the *V*_max_. The *V*_max_ for the liver and tissue are 100.08 and 39.27, respectively. Both the liver and tissue have the same *K_m_* = 1.4 and *K_i_* = 1.4, which is from [24].

| $V_{\mathrm{DNMT}}= \frac{V_{max}[SAM]}{K_{m} \left( 1+\frac{\left[ SAH \right]}{K_{i}} \right)+ \left[ SAM \right]}$ |
| --- |

**SAHH:** Both factors of the SAHH reaction are standard Michaelis-Menten with positive direction from SAH to Hcy and the negative direction from Hcy to SAH. The kinetic constants for SAH in the liver
are $V_{max}^{SAH}$= 480, and $K_{m}^{SAH}$= 6.5 and for the tissue are $V_{max}^{SAH}$ = 310.40, and $K_{m}^{SAH}$= 6.5. The kinetic constants for Hcy in the liver are $V_{max}^{Hcy}$ = 6568.5 and in the tissue $V_{max}^{Hcy}$ = 9513.0, and the Km for both compartments is $K_{m}^{Hcy}$= 150. Justification for the *K_m_* values can be found in [4].

| $V_{\mathrm{SAHH}}= \frac{V_{max}^{SAH}[SAH]}{K_{m}^{SAH} +[SAH]} - \frac{V_{max}^{Hcy}[Hcy]}{K_{m}^{Hcy}+[Hcy]}$ |
| --- |

**BHMT:** The kinetics of BHMT are Michaelis-Menten with the parameters *K_m_* = 12, and
*V*_max_ = 239.3 μM/h [25,26]. The form of the inhibition of BHMT by SAM was derived by non-linear regression on the data of [27] and scaled so that it equals approximately 1 when the SAM and SAH concentrations have their normal steady-state values. The last factor represents the inhibition of BHMT by oxidative stress, see the discussion below. *K_i_* = 0.01 μM is the inhibition constant.

| $V_{\mathrm{BHMT}}= e^{-\left( 0.0021\left( \left[ \mathrm{SAM} \right]+\left[ \mathrm{SAH} \right] \right) \right)}e^{+(0.0021\left( 71.28 \right))}\left( \frac{V_{\max}[Hcy]}{K_{m}+[Hcy]} \right)\left( \frac{K_{i}+{[H_{2}O_{2}]}_{norm}}{K_{i}+[H_{2}O_{2}]} \right)$ |
| --- |

**MS:** Both factors of the MS reaction are standard Michaelis-Menten. The *V*_max_ in the tissue is 15049 μM/h and the liver is *V*_max_ =1421.9 μM/h, and $K_{m}^{Hcy}$= 1 μM [28], and $K_{m}^{5mTHF}$= 25 μM [29,30]. The last factor represents the inhibition of MS by oxidative stress with a *K_i_* = 0.01, see the discussion below.

| $V_{\mathrm{MS}}= \left( \frac{V_{max}[Hcy]}{K_{m}^{Hcy}+[Hcy]} \right)\left( \frac{[5mTHF]}{K_{m}^{5mTHF}+ [5mTHF]} \right)\left( \frac{K_{i}+{[H_{2}O_{2}]}_{norm}}{K_{i}+[H_{2}O_{2}]} \right)$ |
| --- |

**CBS:** The kinetics of CBS is standard Michaelis-Menten where the *V*_max_ in the liver and tissue are 31740 μM/h and 3174 μM/h, respectively. $K_{m}^{Hcy}$ = 1000 μM is taken from [31]. The form of the activation of CBS by SAM and SAH was derived by non-linear regression on the data in [32] and [33] where A = 94 in the liver and A = 35 in the tissue and is scaled so that it equals 1 when the SAM and SAH concentrations are at steady state. The last factor represents the activation of CBS by oxidative stress
with *K_a_* = 0.035, see the discussion below.

| $V_{\mathrm{CBS}}=\left( \frac{V_{max}[Hcy]}{K_{m}^{Hcy}+[Hcy]} \right)\frac{\left( \frac{\left( 1.2 \right)}{(30 + {(\left[ SAM \right]+ \left[ SAH \right]))}^{2}+1} \right)}{\left( \frac{\left( 1.2 \right)}{(30 + {A)}^{2}+1} \right)}\left( \frac{K_{a}+[H_{2}O_{2}]}{K_{a}+{[H_{2}O_{2}]}_{norm}} \right)$ |
| --- |

*C.3. Transport kinetics*

We now discuss the metabolite transport between compartments. Depending on the metabolite being transported, different kinetic equations were used. The parameters are given in Table S6.

**Met and Hcy transport**: The general formula for Met, and Hcy kinetics transport is taken to be

| $\text{V}=\frac{V_{\max}[metabolite]}{K_{m}+[metabolite]}$ |
| --- |

The transport kinetics of Met and Hcy into and out of a compartment are Michaelis-Menten, and the direction of transport is indicated by the subscript of *V*. For example, the transport of Met from the plasma to liver is notated as *V*_pMetl_, and from the liver to the plasma as *V*_lMetp_. The molecular similarities of Hcy and Met allow for movement into or out of cells by multiple cysteine transport systems [34,35]. *K_m_* values for Met transport ranges from 2 to 3000 µM depending on the transport system being used [36], while *K_m_* for Hcy transport ranges from 19 to 1000 µM [37]

**SAM and SAH transport:** SAM and SAH transport is taken to be mass action$.$ In accordance with the literature, we assume that SAM and SAH are only exported from cells into the plasma but are not taken up by cells from the plasma [38–40]. The removal of SAM and SAH from the body is thought to occur through urine [41,42].

**5mTHF transport**: 5mTHF is the most abundant folate found in the plasma [43]. Transport of 5mTHF into and out of a cell occurs through receptor mediated endocytosis, reduced folate-carrier mediated systems, ATP-dependent export and passive diffusion [44].

We use two general formulas for kinetics of folate transport. The first formula is Michaelis-Menten:

| $\text{V}=\frac{V_{\max}\left[ p5mTHF \right]}{K_{m}+\left[ p5mTHF \right]}$ |
| --- |

The subscript of *V* indicates the direction of transport. For instance transport of 5mTHF from the plasma to the liver is indicated by the notation *V*_pfoll_. *K_m_* values of folate uptake into cells range
from 0.66 to 0.76 μM [45,46].

Over supplementation to folate has been shown to decrease folate uptake in cells and has been linked to decreased expression of the RFC [47–49]. We modeled the expression change of RFC by making the rate of transport dependant on the [p5mTHF].

| V_max,tissue_ = 0.094 [p5mTHF]^(−0.7)  V_max,liver_ = 0.28 [p5mTHF]^(−0.7) |
| --- |

Additionally, the model contains bi-directional folate diffusion between liver and plasma and tissue and plasma.

| $V=d\left( \left[ c5mTHF \right]-\left[ p5mTHF \right] \right)$ |
| --- |

where *d* is a rate constant, and c indicates the compartment under consideration (liver or peripheral tissue). The subscript of *V* likewise indicates transport between the relevant compartments. For example, the diffusion of 5mTHF into and out of the liver is represented by *V*_ldiff5mTHF_.

**Table S6.** Parameter values for transport kinetics.

| **Reaction** | **Parameter** | **Model Value** | **Reaction** | **Parameter** | **Model Value** |
| --- | --- | --- | --- | --- | --- |
| *V*_pHcyl_ |  |  | *V*_pMetl_ |  |  |
|  | *K_m_* | 50 |  | *K_m_* | 100 |
|  | *V*_max_ | 121.93 |  | *V*_max_ | 406 |
| *V*_lHcyp_ |  |  | *V*_lMetp_ |  |  |
|  | *K_m_* | 50 |  | *K_m_* | 100 |
|  | *V*_max_ | 44 |  | *V*_max_ | 406 |

**Table S6.** *Cont*.

| *V*_pHcyt_ |  |  | *V*_pMett_ |  |  |
| --- | --- | --- | --- | --- | --- |
|  | *K_m_* | 50 |  | *K_m_* | 100 |
|  | *V*_max_ | 5.05 |  | *V*_max_ | 16000 |
| *V*_tHcyp_ |  |  | *V*_tMetp_ |  |  |
|  | *K_m_* | 50 |  | *K_m_* | 100 |
|  | *V*_max_ | 526.64 |  | *V*_max_ | 1510 |
| *V*_lSAMp_ |  |  | *V*_lSAHp_ |  |  |
|  | *k*_lSAMp_ | 0.00095 |  | *k*_lSAHp_ | 0.0009 |
| *V*_tSAMp_ |  |  | *V*_tSAHp_ |  |  |
|  | *k*_tSAMp_ | 0.0035 |  | *k*_tSAHp_ | 0.0007 |
| *V*_p5mTHFl_ |  |  | *V*_p5mTHFt_ |  |  |
|  | *K_m_* | 0.7 |  | *K_m_* | 0.7 |
| *V*_ldiff5mTHF_ |  |  | *V*_tdiff5mTHF_ |  |  |
|  | *d*_ldiff_ | 0.000012 |  | *d*_tdiff_ | 0.00001 |

**Part D. Input and Removal Rate of Metabolites**

*D.1. Input Rates (µM/h)*

**Folate:** After establishing the metabolic kinetics and transport kinetics for each compartment we found that a 5mTHF input rate into the plasma compartment of 0.0046 μmol L^−1^ h^−1^ gave rise to tissue folate concentration of 0.45 μM and a liver concentration of 21.03 μM. These values are close to the observed pre-fortification mean erythrocyte folate concentrations corresponding to an estimated average dietary folate intake of about 200 μg/day. When we increased mean folate input 1.5-fold, we obtained a steady-state tissue folate concentration of 0.599 μM, which matches the mean
post-fortification erythrocyte folate concentration, corresponding to an estimated average dietary intake of about 300 μg/day.

In the pulsatile folate load test described in Section 3.1, the folate input to the plasma was:

| FolateIn = 0.0046(1 + 5*t*/(1 + (0.2)*t*^4^)) |
| --- |

**Methionine:** Methionine input in the model is 100 µM per h except in the case of a methionine load test where the methionine input is:

| Lmetin = 100(1 + 5*t*^2^/(1 + (0.1)*t*^4^)) |
| --- |

D.2. Removal Rates (µM/h)

The removal of 5mTHF, Met, SAM and SAH from the plasma to the urine follows the formula

| $V=k[c\_metabolite]$ |
| --- |

where k is a constant that was calculated so that the rate of removal of a metabolite from a compartment accurately reflects experimental data, and c is the compartment the metabolite is currently occupying. The removal of a metabolite is unidirectional and expressed by a linear equation, which is indicated by its transport velocity. For example, the removal of methionine from the plasma is represented by *V*_pmetu_.

A major route of metabolites removal from the body is through filtration of the plasma by the kidney and ultimate loss of the metabolite via urine excretion. Our model does not contain a kidney compartment so loss of a metabolite occurs directly by the removal of the metabolite from the plasma. Additionally, our model assumes that loss of urine is 1 L/per 24 h, which is within the normal range of daily human urine loss [50].

Catabolism by liver and tissue removes most folate from the body, and approximately 1% of folate removal occurs through urine [51]. Our model accounts for folate catabolism in the liver and tissue as well as loss through urine by linear catabolism of THF.

**Hcy removal:** We now discuss removal of Hcy from the plasma which is expressed by the formula

| $V_{\mathrm{pHcyu}}=\left( 0.48e^{\left( 0.125*\left[ pHCY \right] \right)} \right)*0.073*\left[ \mathrm{pHcy} \right]- \frac{V_{\max}[uHcy]}{K_{m}+[uHcy]}$ |
| --- |

where the prefixes *p* and *u* stand for the concentration of Hcy in the plasma and the urine. Most Hcy is reabsorbed by the kidney, which allows only 1%–2% of it to be removed daily in urine [50,52]. For the purpose of these calculations we assume that plasma and urine compartments have the same volume and that the transports occur within the kidney tubules and associated capillaries. The kinetics are exponential for Hcy going from the plasma and into the kidney-urine compartment, with the rate of removal of Hcy from the plasma dependent on the concentration of Hcy. The kinetics is Michaelis-Menten for Hcy going from the kidney-urine compartment back into the plasma, with *V*_max_ = 0.5, and *K_m_* = 1. The removal rate is 0.009 µM/h at steady state in our model with the normal parameters.

**Table S7.** Rate constants for removal of metabolites by catabolism and excretion, and the rate of metabolite loss from the various compartments.

| **Compartment** | **Metabolite removed** | **Removal constant** | | **Rate of removal at steady-state (µM/h)** | | **Ref.** |
| --- | --- | --- | --- | --- | --- | --- |
|  |  | **name** | **value (h^−1^)** | **model** | **experiment** |  |
| Plasma | Met | k_pMetu_ | 0.05 | 1.65 | 1.7 | [53] |
|  | SAM | k_pSAMu_ | 9 | 0.81 | 0.42 | [42] |
|  | SAH | k_pSAHu_ | 1.4 | 0.03 | 0.02 | [42] |
|  | 5mTHF | k_pfolateu_ | 0.01 | 0.0001 | 0.00025 | [54] |
| Liver | THF | k_lfolate_ | 0.0007 | 0.005 |  |  |
| Tissue | THF | k_tfolate_ | 0.0008 | 0.0001 |  |  |

**Figure S2.** **Relationships among plasma metabolites, tissue metabolites and tissue enzyme activities.** Each square contains 10,000 points, representing virtual individuals in the database in DuncanPopulationData.xls. The red ellipses encompass about 95% of the data in each square. Many of the relationships are non-linear. Names of metabolites and enzymes are on the diagonal. Columns represent variation of the variable along the *x*-axis, and the *y*-axes in the column show the variation of the different row-variables.

**
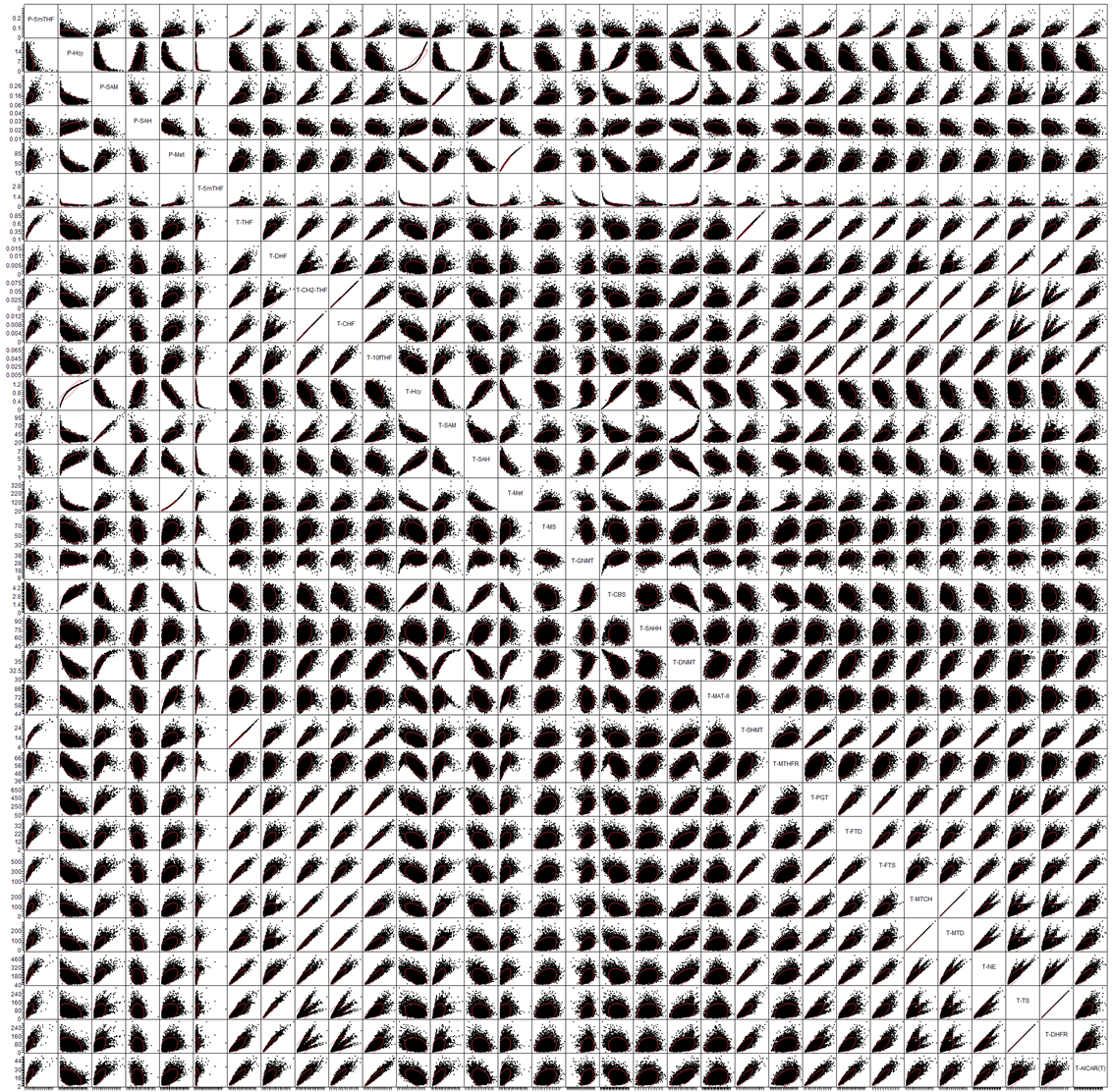
**

**Figure S3.** **Relationships among plasma metabolites, liver metabolites and liver enzyme activities.** Each square contains 10,000 points, representing virtual individuals in the database in DuncanPopulationData.xls. The red ellipses encompass about 95% of the data in each square. Many of the relationships are non-linear. Names of metabolites and enzymes are on the diagonal. Columns represent variation of the variable along the *x*-axis, and the *y*-axes in the column show the variation of the different row-variables. The correlations between plasma and liver variables are weaker than those for plasma and tissue variables shown in Figure S2.

**
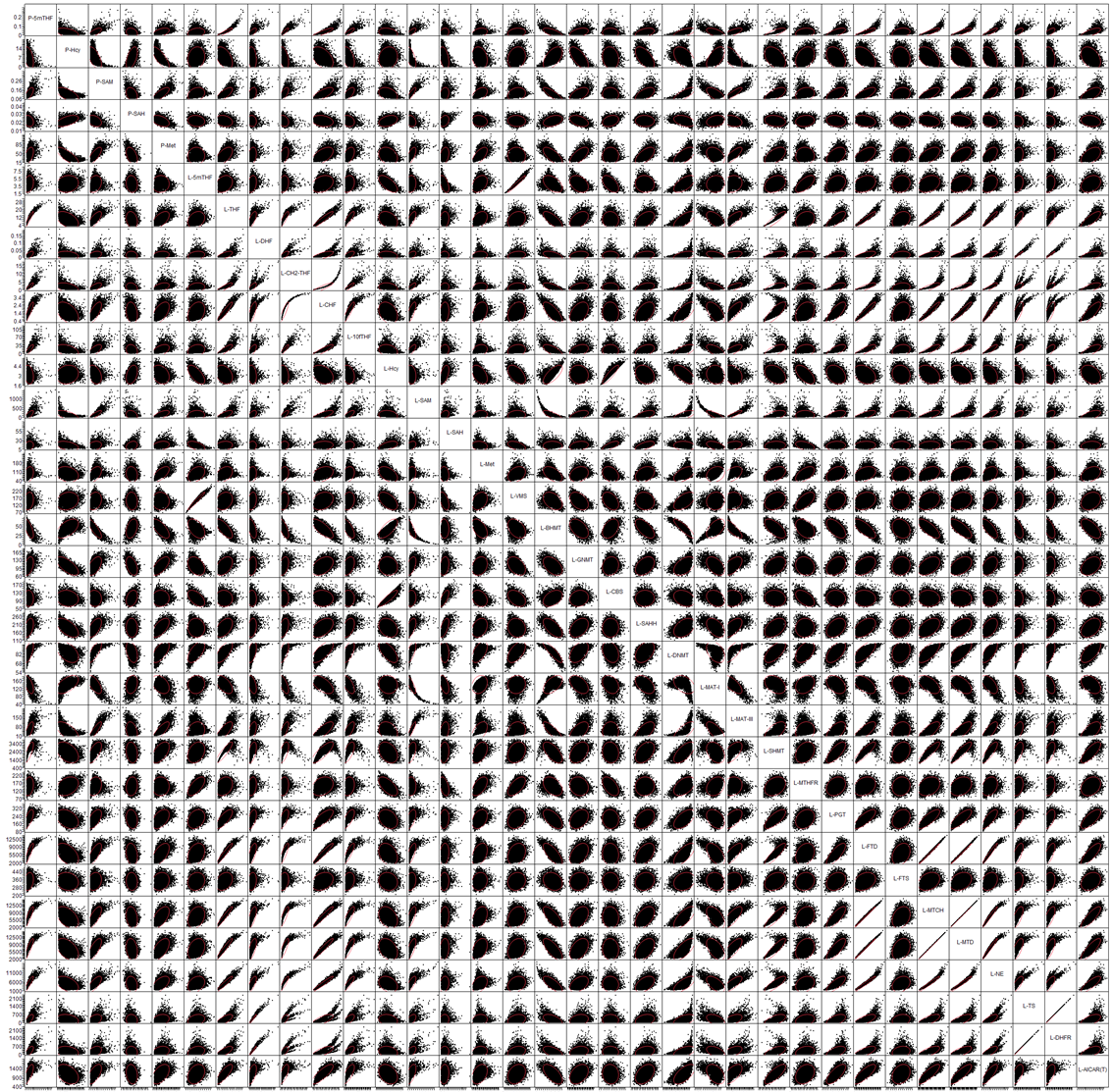
**

**References**

1. Duncan, T.; Reed, M.; Nijhout, H. The relationship between intracellular and plasma levels of folate and metabolites in the methionine cycle: A model. *Mol. Nutr. Food Res.* **2013**, *57*, 626–638.
2. Nijhout, H.; Reed, M.; Lam, S.; Shane, B.; Gregory, J.I.; Ulrich, C. In silico experimentation with a model of hepatic mitochondrial folate metabolism. *Theor.* *Biol. Med.* *Model.* **2006**, *3*, doi:10.1186/1742-4682-3-40.
3. Nijhout, H.F.; Reed, M.C.; Budu, P.; Ulrich, C.M. A mathematical model of the folate cycle—New insights into folate homeostasis. *J. Biol. Chem.* **2004**, *279*, 55008–55016.
4. Reed, M.; Thomas, R.; Pavisic, J.; James, S.; Ulrich, C.; Nijhout, H. A mathematical model of glutathione metabolism. *Theor. Biol. Med. Model.* **2008**, 5, doi:10.1186/1742-4682-5-8.
5. Ulrich, C.M.; Neuhouser, M.; Liu, A.Y.; Boynton, A.; Gregory, J.F., III; Shane, B.; James, S.J.; Reed, M.C.; Nijhout, H.F. Mathematical modeling of folate metabolism: Predicted effects of genetic polymorphisms on mechanisms and biomarkers relevant to carcinogenesis. *Cancer Epidemiol.* *Biomark. Prev.* **2008**, *17*, 1822–1831.
6. Lindenbaum, J.; Allen, R.H. Clincal Spectrum and Diagnosis of Folate Deficiency. In *Folate in Health and Disease*, Bailey, L., Ed.; Marcel Dekker, Inc.: New York, NY, USA, 1995; pp. 43–74.
7. Ganji, V.; Kafai, M. Trends in serum folate, rbc folate, and circulating total homocysteine concentrations in the united states: Analysis of data from national health and nutrition examination surveys, 1988–1994, 1999–2000, and 2001–2002. *J. Nutr.* **2006**, *136*, 153–158.
8. Loehrer, F.; Angst, C.; Brunner, F.; Haefeli, W.; Fowler, B. Evidence for disturbed
   *S*-adenosylmethionine: *S*-adenosylhomocysteine ratio in patients with end-stage renal failure: A cause for disturbed methylation reactions? *Nephrol. Dial. Transplant.* **1998**, *13*, 656–661.
9. Brouwer, I.; van Dusseldorp, M.; Duran, M.; Thomas, C.; Hautvast, J.; Eskes, T.;
   Steegers-Theunissen, R. Low-dose folic acid supplementation does not influence plasma methionine concentrations in young non-pregnant women. *Br. J. Nutr.* **1999**, *82*, 85–89.
10. Fowler, B. Transport and Tissue Distribution of Homocysteine and Related *S*-Adensoyl Compounds. In *Homocysteine in Health and Disease*, Carmel, R., Jacobsen, D., Eds.; Cambridge University Press: Cambridge, UK, 2001; pp. 163–175.
11. Hoppner, K.; Lampi, B. Folate levels in human-liver autopsies in canada. *Am. J. Clin. Nutr.* **1980**, *33*, 862–864.
12. Agrimi, G.; Di Noia, M.; Marobbio, C.; Fiermonte, G.; Lasorsa, F.; Palmieri, F. Identification of the human mitochondrial *S*-adenosylmethionine transporter: Bacterial expression, reconstitution, functional characterization and tissue distribution. *Biochem. J.* **2004**, *379*, 183–190.
13. Finkelstein, J.; Martin, J. Methionine metabolism in mammals. Distribution of homocysteine between competing pathways. *J. Biol. Chem.* **1984**, *259*, 9508–9513.
14. Brown, R.; Delp, M.; Lindstedt, S.; Rhomberg, L.; Beliles, R. Physiological parameter values for physiologically based pharmacokinetic models. *Toxicol.* *Ind. Health* **1997**, *13*, 407–484.
15. Deplancke, B.; Gaskins, H. Redox control of the transsulfuration and glutathione biosynthesis pathways. *Curr.* *Opin. Clin. Nutr.* *Metab. Care* **2002**, *5*, 85–92.
16. Mosharov, E.; Cranford, M.; Banerjee, R. The quantitatively important relationship between homocysteine metabolism and glutathione synthesis by the transsulfuration pathway and its regulation by redox changes. *Biochemistry* **2000**, *39*, 13005–13011.
17. Corrales, F.; Ruiz, F.; Mato, J. *In vivo* regulation by glutathione of methionine adenosyltransferase *S*-nitrosylation in rat liver. *J. Hepatol.* **1999**, *31*, 887–894.
18. Pajares, M.; Duran, C.; Corrales, F.; Pliego, M.; Mato, J. Modulation of rat liver
    *S*-adenosylmethionine synthetase activity by glutathione. *J. Biol. Chem.* **1992**, *267*, 17598–17605.
19. Sullivan, D.; Hoffman, J. Fractionation and kinetic properties of rat liver and kidney methionine adenosyltransferase isozymes. *Biochemistry* **1983**, *22*, 1636–1641.
20. Del Pino, M.; Corrales, F.; Mato, J. Hysteretic behavior of methionine adenosyltransferase
    III––Methionine switches between two conformations of the enzyme with different specific activity. *J. Biol. Chem.* **2000**, *275*, 23476–23482.
21. Ogawa, H.; Fujioka, M. Purification and properties of glycine *N*-methyltransferase from rat liver.
    *J. Biol. Chem.* **1982**, *257*, 3447–3452.
22. Kim, D.; Huang, T.; Schirch, D.; Schirch, V. Properties of tetrahydropteroylpentaglutamate bound to 10-formyltetrahydrofolate dehydrogenase. *Biochemistry* **1996**, *35*, 15772–15783.
23. Yeo, E.; Wagner, C. Purification and properties of pancreatic glycine *N*-methyltransferase. *J. Biol. Chem.* **1992**, *267*, 24669–24674.
24. Flynn, J.; Reich, N. Murine DNA (cytosine-5-)-methyltransferase: Steady-state and substrate trapping analyses of the kinetic mechanism. *Biochemistry* **1998**, *37*, 15162–15169.
25. Finkelstein, J.; Harris, B.; Kyle, W. Methionine metabolism in mammals: Kinetic study of
    betaine-homocysteine methyltransferase. *Arch. Biochem. Biophys.* **1972**, *153*, 320–324.
26. Skiba, W.; Taylor, M.; Wells, M.; Mangum, J.; Awad, W. Human hepatic methionine biosynthesis. Purification and characterization of betaine-homocysteine *S*-methyltransferase. *J. Biol. Chem.* **1982**, *257*, 4944–4948.
27. Finkelstein, J.; Martin, J. Inactivation of betaine-homocysteine methyltransferase by adenosylmethionine and adenosylethionine. *Biochem. Biophys. Res. Commun.* **1984**, *118*, 14–19.
28. Banerjee, R.; Chen, Z.; Gulati, S. Methionine synthase from pig liver. *Methods Enzymol.* **1997**, *281*, 189–196.
29. Banerjee, R.; Frasca, V.; Ballou, D.; Matthews, R. Participation of cob(i)alamin in the reaction catalyzed by methionine synthase from escherichia coli: A steady-state and rapid reaction kinetic analysis. *Biochemistry* **1990**, *29*, 11101–11109.
30. Finkelstein, J.; Martin, J. Methionine metabolism in mammals. Adaptation to methionine excess.
    *J. Biol. Chem.* **1986**, *261*, 1582–1587.
31. Finkelstein, J. Regulation of Homocysteine Metabolism. In *Homocysteine in Health and Disease*, Carmel, R., Jacobsen, D.W., Eds.; Cambridge University Press: Cambridge, UK, 2001; pp. 92–99.
32. Janosik, M.; Kery, V.; Gaustadnes, M.; Maclean, K.; Kraus, J. Regulation of human cystathionine
    β-synthase by *S*-adenosyl-l-methionine: Evidence for two catalytically active conformations involving an autoinhibitory domain in the *C*-terminal region. *Biochemistry* **2001**, *40*, 10625–10633.
33. Kluijtmans, L.; Boers, G.; Stevens, E.; Renier, W.; Kraus, J.; Trijbels, F.; van den Heuvel, L.; Blom, H. Defective cystathionine β-synthase regulation by *S*-adenosylmethionine in a partially pyridoxine responsive homocystinuria patient. *J. Clin. Investig.* **1996**, *98*, 285–289.
34. Ewadh, M.; Tudball, N.; Rose, A. Homocysteine uptake by human umbilical vein endothelial cells in culture. *Biochim. Biophys. Acta* **1990**, *1054*, 263–266.
35. Korendyaseva, T.; Martinove, M.; Dudchenko, A.; Vitvitsky, V. Distribution of methionine between cells and incubation medium in suspension of rat hepatocytes. *Amino Acids* **2010**, *39*, 1281–1289.
36. Soriano-Garcia, J.; Torras-Llort, M.; Ferrer, R.; Moreto, M. Multiple pathways for l-methionine transport in brush-border membrane vesicles from chicken jejunum. *J. Physiol.* **1998**, *509*, 527–539.
37. Budy, B.; O’Neill, R.; DiBello, P.; Sengupta, S.; Jacobsen, D. Homocysteine transport by human aortic endothelial cells: Identification and properties of import systems. *Arch. Biochem. Biophys.* **2006**, *446*, 119–130.
38. Greenberg, M.; Chaffee, S.; Hershfield, M. Basis for resistance to 3-deazaaristeromycin, an inhibitor of *S*-adenosylhomocysteine hydrolase, in human b-lymphoblasts. *J. Biol. Chem.* **1989**, *264*, 795–803.
39. Hoffman, D.; Marion, D.; Cornatzer, W.; Duerre, J. *S*-adenosylmethionine and *S*-adenosylhomocystein metabolism in isolated rat liver. Effects of l-methionine, l-homocystein, and adenosine. *J. Biol. Chem.* **1980**, *255*, 822–827.
40. Bontemps, F.; VandenBerghe, G. Metabolism of exogenous *S*-adenosylmethionine in isolated rat hepatocyte suspensions: Methylation of plasma-membrane phospholipids without intracellular uptake. *Biochem. J* **1997**, *327*, 383–389.
41. Duerre, J.; Miller, C.; Reams, G. Metabolism of *S*-adenosyl-l-homocysteine *in vivo* by the rat.
    *J. Biol. Chem.* **1969**, *244*, 107–111.
42. Stabler, S.; Allen, R. Quantification of serum and urinary *S*-adenosylmethionine and
    *S*-adenosylhomocysteine by stable-isotope-dilution liquid chromatography-mass spectrometry. *Clin. Chem.y* **2004**, *50*, 365–372.
43. Selhub, J.; Jacques, P.F.; Wilson, P.W.F.; Rush, D.; Rosenberg, I.H. Vitamin status and intake as primary determinants of homocysteinemia in an elderly population. *JAMA* **1993**, *270*, 2693–2698.
44. Suh, J.; Herbig, A.; Stover, P. New perspectives on folate catabolism. *Annu. Rev. Nutr.* **2001**, *21*, 255–282.
45. Nabokina, S.; Ma, T.; Said, H. Mechanism and regulation of folate uptake by human pancreatic epithelial mia paca-2 cells. *Am. J. Physiol. Cell Physiol.* **2004**, *287*, C142–C148.
46. Nguyen, T.; Dyer, D.; Dunning, D.; Rubin, S.; Grant, K.; Said, H. Human intestinal folate transport: Cloning, expression, and distribution of complementary rna. *Gastroenterology* **1997**, *112*, 783–791.
47. Lowering blood homocysteine with folic acid-based supplements: Meta-analysis of randomised trials. *Indian Heart J.* **2000**, *52*, S59–S64.
48. Halsted, C.H.; Villanueva, J.A.; Devlin, A.M.; Niemela, O.; Parkkila, S.; Garrow, T.A.; Wallock, L.M.; Shigenaga, M.K.; Melnyk, S.; James, S.J. Folate deficiency disturbs hepatic methionine metabolism and promotes liver injury in the ethanol-fed micropig. *Proc. Natl. Acad. Sci. USA* **2002**, *99*, 10072–10077.
49. Caudill, M.A.; Wang, J.C.; Melnyk, S.; Pogribny, I.P.; Jernigan, S.; Collins, M.D.; Santos-Guzman, J.; Swendseid, M.E.; Cogger, E.A.; James, S.J. Intracellular *S*-adenosylhomocysteine concentrations predict global DNA hypomethylation in tissues of methyl-deficient cystathionine β-synthase heterozygous mice. *J. Nutr.* **2001**, *131*, 2811–2818.
50. Chwatko, G.; Jakubowski, H. Urinary excretion of homocysteine-thiolactone in humans. *Clin. Chem.* **2005**, *51*, 408–415.
51. InsituteofMedicine. *Dietary Reference Intak*; National Academy Press: Washington, DC, USA, 1998.
52. Refsum, H.; Helland, S.; Ueland, P. Radioenzymic determination of homocysteine in plasma and urine. *Clin. Chem.* **1985**, *31*, 624–628.
53. Stipanuk, M. Sulfur amino acid metabolism: Pathways for production and removal of homocysteine and cysteine. *Annu. Rev. Nutr.* **2004**, *24*, 539–577.
54. Gregory, J.; Williamson, J.; Bailey, L.; Toth, J. Urinary excretion of h-2(4) folate by nonpregnant women following a single oral dose of h-2(4) folic acid is a functional index of folate nutritional status. *J. Nutr.* **1998**, *128*, 1907–1912.
